# The Nerve Growth Factor Receptor CD271 Is Crucial to Maintain Tumorigenicity and Stem-Like Properties of Melanoma Cells

**DOI:** 10.1371/journal.pone.0092596

**Published:** 2014-05-05

**Authors:** Torben Redmer, Yvonne Welte, Diana Behrens, Iduna Fichtner, Dorothea Przybilla, Wasco Wruck, Marie-Laure Yaspo, Hans Lehrach, Reinhold Schäfer, Christian R. A. Regenbrecht

**Affiliations:** 1 Institute of Pathology - University Hospital Berlin, Berlin, Germany; 2 Experimental Pharmacology & Oncology Berlin-Buch GmbH, Berlin, Germany; 3 Laboratory of Functional Genomics (LFGC) - University Hospital Berlin, Berlin, Germany; 4 Max-Planck Institute for Molecular Genetics, Berlin, Germany; 5 Comprehensive Cancer Center Charité - University Hospital Berlin, Berlin, Germany; City of Hope, United States of America

## Abstract

**Background:**

Large-scale genomic analyses of patient cohorts have revealed extensive heterogeneity between individual tumors, contributing to treatment failure and drug resistance. In malignant melanoma, heterogeneity is thought to arise as a consequence of the differentiation of melanoma-initiating cells that are defined by cell-surface markers like CD271 or CD133.

**Results:**

Here we confirmed that the nerve growth factor receptor (CD271) is a crucial determinant of tumorigenicity, stem-like properties, heterogeneity and plasticity in melanoma cells. Stable shRNA mediated knock-down of CD271 in patient-derived melanoma cells abrogated their tumor-initiating and colony-forming capacity. A genome-wide expression profiling and gene-set enrichment analysis revealed novel connections of CD271 with melanoma-associated genes like CD133 and points to a neural crest stem cell (NCSC) signature lost upon CD271 knock-down. In a meta-analysis we have determined a shared set of 271 differentially regulated genes, linking CD271 to SOX10, a marker that specifies the neural crest. To dissect the connection of CD271 and CD133 we have analyzed 10 patient-derived melanoma-cell strains for cell-surface expression of both markers compared to established cell lines MeWo and A375. We found CD271^+^ cells in the majority of cell strains analyzed as well as in a set of 16 different patient-derived melanoma metastases. Strikingly, only 2/12 cell strains harbored a CD133^+^ sub-set that in addition comprised a fraction of cells of a CD271^+^/CD133^+^ phenotype. Those cells were found in the label-retaining fraction and *in vitro* deduced from CD271^+^ but not CD271 knock-down cells.

**Conclusions:**

Our present study provides a deeper insight into the regulation of melanoma cell properties and points CD271 out as a regulator of several melanoma-associated genes. Further, our data strongly suggest that CD271 is a crucial determinant of stem-like properties of melanoma cells like colony-formation and tumorigenicity.

## Introduction

Malignant melanomas originate from the oncogenic transformation of melanocytes [1] the pigment cells of the skin and the eyes. These among others stem from a multipotent neural crest stem cell (NCSC) that expresses the nerve growth factor receptor CD271 and the SRY-box transcription factor SOX10 [2]. The latter acts in concert with a well-defined set of growth factors and inhibitors (reviewed in [3]) to trigger the derivation of melanocytes from the NCSC. Recent studies may evidence that the sustained expression of CD271 during melanocyte development may support the process of melanoma formation [4]. Consistently, CD271 was recognized recently as a crucial molecule that drives melanoma initiation and metastasis by a yet unknown mechanism [5] thus endowing melanoma cells with stem-like properties. Stem-like features of melanoma cells were additionally associated with the presence of the glioma and neural stem cell marker CD133 (also known as PROM1) [6], [7]. CD133 expression is correlated with a high tumorigenic and metastatic potential of melanoma cells and facilitates cell motility [8]. Like CD271 and CD133 in melanoma-initiating cells, SOX10 expression maintains the multipotent phenotype of NCSCs [2] and the tumorigenic and proliferative capacity of melanoma cells [9].

Like their stem cell counterparts in normal tissue, tumor-initiating cells are capable to self-renew [10] by asymmetric cell division resulting in the formation of a tumor-initiating cell and differentiated progeny which have lost the tumor-initiating potential of the mother cell [11]. In malignant melanomas this progeny is characterized by either the melanoma antigens HMB45 and MART-1 [12], the microphthalmia-associated transcription factor (MITF) or the melanin synthesis controlling enzyme tyrosinase (TYR). However, besides asymmetric cell division, label-retention and colony-formation as well as a high cellular plasticity are determinants of stem-like cells. Cellular plasticity of tumor-initiating cells represents a proposed mechanism that leads to intra-tumor heterogeneity as they are thought to be capable to enter, exit and to re-enter a stem-cell state while changing their phenotype defined by expression of cell surface markers like CD271 or ABCB5 [13]. The cellular heterogeneity leads to variations of cancer cells, residing in a tumor along with stromal elements that collectively form the microenvironment. Since solid tumors can be composed of a variety of clones or subpopulations of cancer cells, these cells may differ among themselves in many properties, such as growth rate, production and expression of cell surface markers or sensitivity to therapeutics (chemo, biologic, radiation).

However, the implementation of markers of melanoma-initiating cells to determine stem-like properties as well as their role within a melanoma-associated network is elusive. Here we uncover the significance of CD271 expression for melanoma cell tumorigenicity and plasticity and demonstrate a CD271-dependend regulation of the neural crest specifier SOX10 and the connection to CD133. Further we show that both CD271^+^ and CD133^+^ cells exhibit plasticity allowing the transition of CD271^+^ cells to adopt a CD271^+^/CD133^+^ phenotype and the transition of CD133^−^ cells to form CD133^+^ tumors.

## Results

### Expression of CD271 is crucial for the tumorigenic growth of melanoma cells

**Figure 1:**
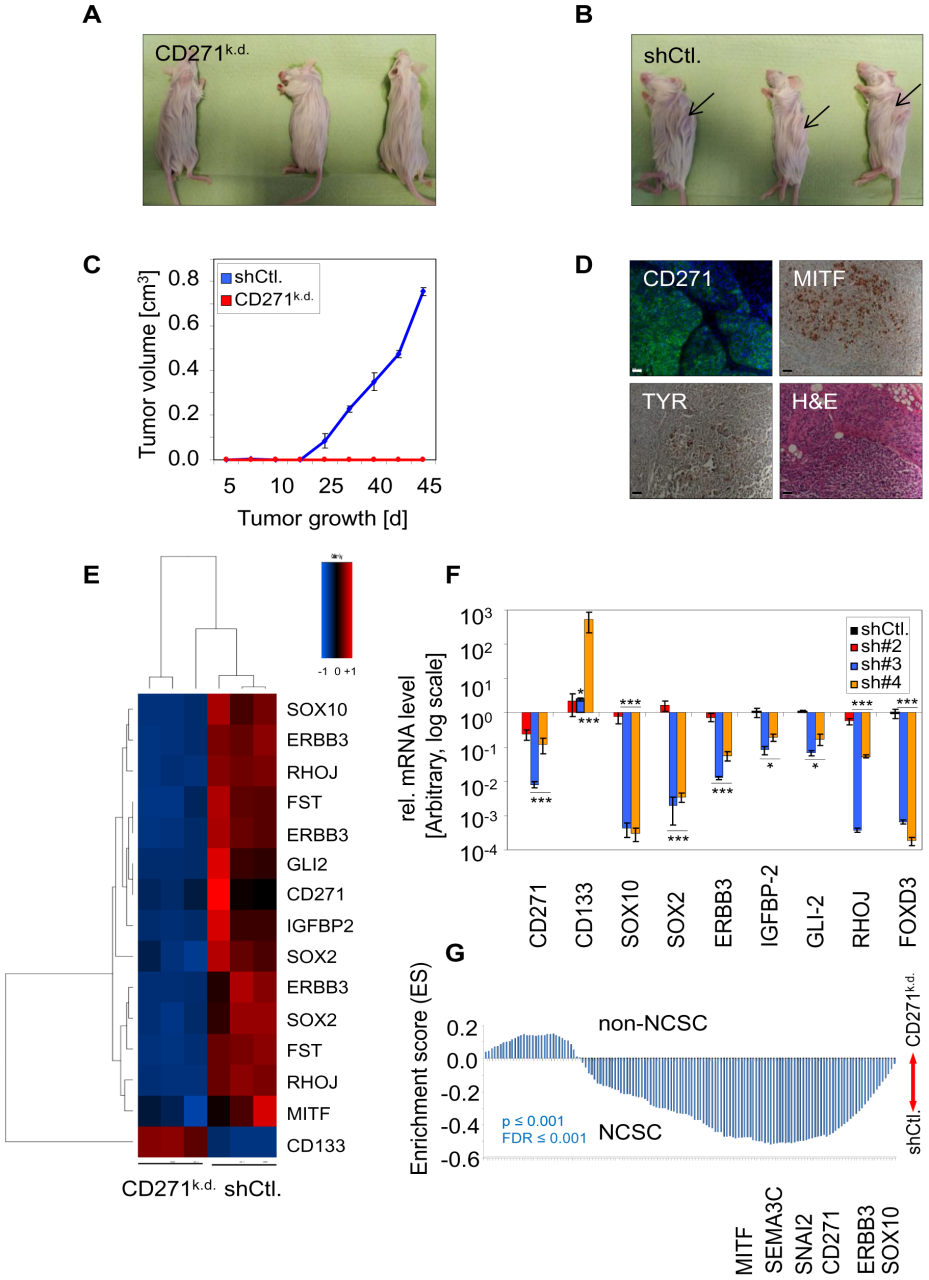
Expression of CD271 in melanoma cells is a crucial determinant of proliferation and tumorigenicity. (**A**) Absence of subcutaneous tumors 45 days after injection of 1×10^6^ CD271 knock-down (CD271^k.d.^) cells into NSG mice. Cells were stably transfected with shRNA#4. (**B–C**) Simultaneous injection of cells stably transfected with a shRNA control plasmid (shCtl.) led to proper formation of tumors (arrows in B). Tumor growth is shown as mean values ± SD of biological triplicates. (**D**) Tumors of (B) showed expression of CD271, MITF and TYR. H&E indicates tumor histology. (**E**) Heat map of 10 significantly regulated melanoma-associated genes selected from a genome-wide expression profiling. Different splice forms of ras homolog family member J (*RHOJ*), v-Erb-B2 erythroblastic leukemia viral oncogene homolog 3 (*ERBB3*), SRY-box 2 (*SOX2*) and follistatin (*FST*) are included. Independent biological triplicates of either CD271^k.d.^ cells or shCtl. cells were analyzed. Up-regulated or down-regulated genes are indicated in red or blue, respectively. (**F**) Validation of genome-wide expression profiling by qPCR for the regulation of CD271, CD133, SOX10, SOX2, ERBB3, insulin-like growth factor binding protein 2 (*IGFBP-2*), GLI-family zinc finger 2 (*GLI-2*), RHOJ and forkhead-box D3 (*FOXD3*) as an additional gene in patient-derived melanoma cells stably transfected with shRNA plasmids #2, #3 or #4 (CD271^k.d.^). Expression levels of shRNA control (shCtl.) cells and CD271^k.d.^ cells are shown as ΔΔCT values normalized to β-actin and related to shCtl. cells as mean values ± SD of biological triplicates. *p≤0.05; ***p≤0.001 (t-test). The scale is logarithmic (log). (**G**) Comparison of data sets of shCtl. and CD271^k.d.^ cells with data sets of human embryonic stem cell derived neural crest stem cells (NCSC) [50] by gene-set enrichment analysis (GSEA). GSEA revealed the presence of a NCSC-specific gene signature in shCtl. cells that is lost upon CD271 silencing (non-NCSC signature). Genes found in the signature among others are CD271, *ERBB3*, *SOX10*, microphthalmia-associated transcription factor (*MITF*), snail homolog 2 (*SNAI2*) and semaphorin 3C (*SEMA3C*).

Melanoma cells expressing the neural crest stem cell marker CD271 are considered as the crucial cellular compartment capable of tumor initiation and differentiation [14], [15]. To investigate the role of CD271, we performed a stable shRNA-mediated knock-down in a patient-derived melanoma cell strain using different shRNA plasmids (#2, #3 and #4). Silencing of CD271 induced a change in morphology from spindle-shaped to epithelial-like as indicated by a change in the localization of CD166 and led to a strong reduction of proliferation (Figure S1A–B). To investigate the effect of CD271 knock-down on tumorigenicity we injected 1×10^6^ of either shRNA#4 transfected (CD271^k.d.^) cells or cells transfected with a control shRNA (shCtl.) into NSG mice (NOD/SCID mouse strain with a lack in IL2 receptor gamma chain). After six weeks no tumors were observed in CD271^k.d.^ cell recipient mice (Figure 1A), whereas shCtl. cells prompted tumor development (Figure 1B–C). Xenografted tumors displayed expression of CD271 as well as melanoma differentiation markers TYR and MITF (Figure 1D). To identify putative CD271 dependent regulatory mechanisms we compared CD271^k.d.^ cells and shCtl. cells in a genome-wide expression profiling. We identified 68 down-regulated (ratio <0.05fold) and 55 up-regulated genes (ratio >20fold), (Figure S1C). We focused on 10 of the most important and significantly regulated (limma_FDR≤0.005) genes that are known to be crucial for melanoma development. We found 9 genes strongly down-regulated like *SOX10* (31-fold), *ERBB3* (9-fold), *IGFBP2* (7-fold), *RHOJ* (6-fold), *FST* (4-fold), *SOX2* (3-fold), *GLI-2* (3-fold) and *MITF* (1.4-fold) upon CD271 knock-down (Figure 1E). With CD133 differentially expressed, our screen identified another putative marker of melanoma-initiating cells. By qPCR we confirmed the differential expression of a majority of these genes in CD271^k.d.^ cells and prompted an inverse correlation of CD271 and CD133 mRNA expression levels. Increased levels of CD133 mRNA by three different shRNA sequences as well as the strong decrease of *SOX10* and the neural crest marker *FOXD3* [16] was verified (Figure 1F). Immunofluorescence microscopy verified the down-regulation of SOX10 and FOXD3 in the majority of cells (Figure S1D). Western blot for CD271 and SOX10 further confirmed the loss of these markers (Figure S1E). The inverse correlation of CD271 and CD133 was further observed by over-expression of CD271 (CD271^exo^) which led to a significantly decreased level of CD133 (Figure S1F). We also observed the inverse correlation of CD271 and CD133 in MeWo cells. However, in these cells we verified the regulation of some but not all CD271-associated genes e.g. FOXD3 and SOX10 by qPCR and immunofluorescence microscopy, respectively (Figure S2A–B). Further we observed a mutually exclusive expression of SOX10 and CD166 in MeWo cells. Those cells that escaped stable selection following transfection with shRNA#4 retained SOX10 expression (Figure S2C). To elucidate the overall effect of the CD271 knock-down we performed gene-set enrichment analysis (GSEA). GSEA revealed a neural crest stem cell (NCSC) signature of shCtl. cells that was lost by knock-down. The NCSC-signature among others comprised those genes we found strongly down-regulated in CD271^k.d.^ cells like *SOX10*, *ERBB3* and *MITF* but also *SNAI2* and *SEMA3C* (Figure 1G). These data suggest CD271 as a determinant of properties of melanoma-initiating cells and melanoma cells. Commonly, tumor-initiating cells evade apoptosis. Therefore we asked whether down-regulation of CD271 in melanoma cells may affect levels of anti-apoptotic genes like cIAP-1, cIAP-2 and BCL-2 (reviewed in [17]). Indeed, we found intensely decreased levels of these anti-apoptotic genes upon silencing of CD271 (Figure S2D). In addition, we observed an increased frequency of cells with DNA-damage as determined by immunofluorescence microscopy for phosphorylation of histone H2AX (γH2AX) [18], (Figure S2E). However, we did not observe apoptotic cells by cleavage of caspase-3 in knock-down cells (data not shown).

### CD271^+^ and CD133^+^ melanoma cell sub-sets exhibit *in vivo* plasticity

**Figure 2:**
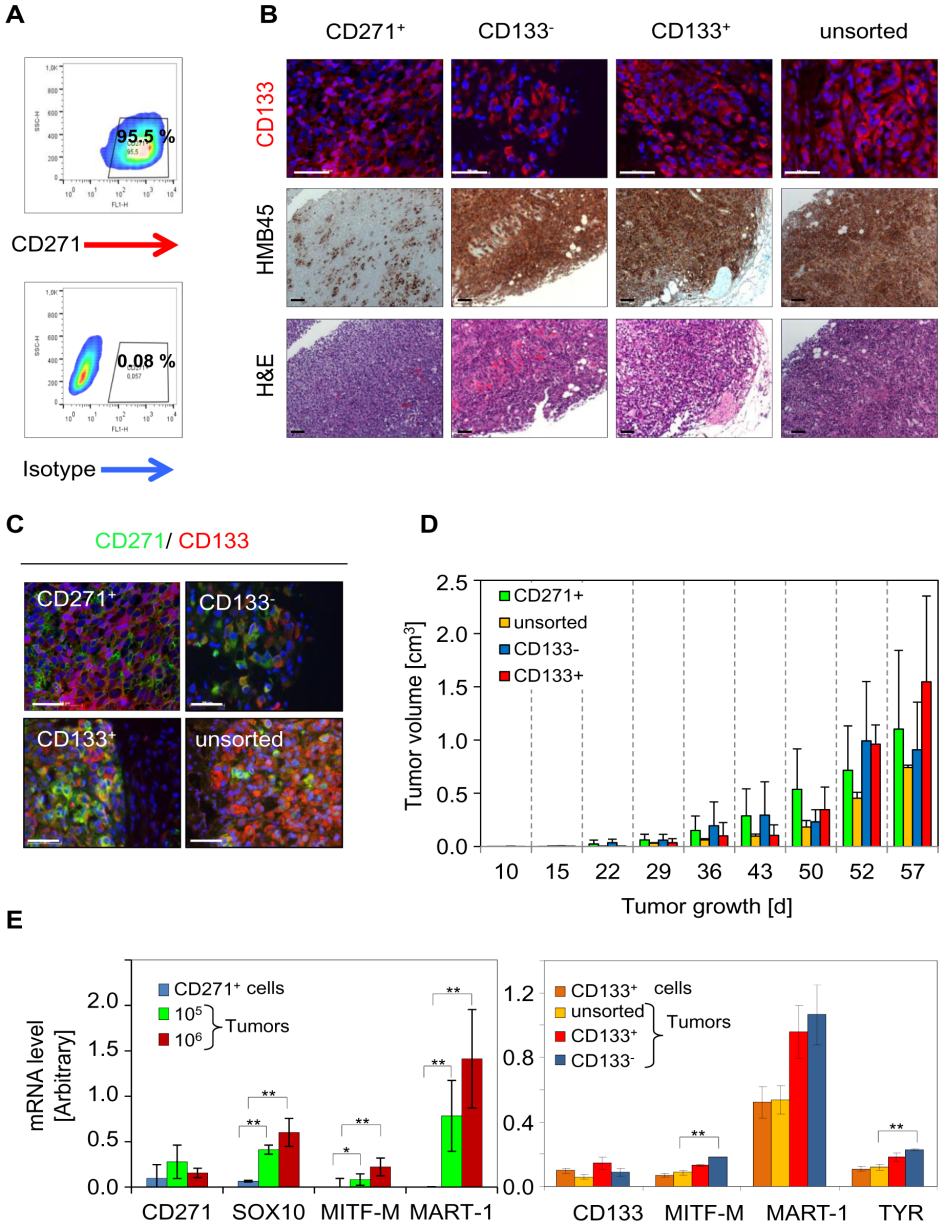
CD271^+^ and CD133^+^ melanoma cells hold comparable tumor-initiating capacities. (**A**) Flow cytometry of enriched CD271^+^ cells show a high content of CD271 expressing cells before transplantation. (**B**) Immunofluorescence analysis and immunohistochemistry on paraffin sections (2 µm) of tumors derived from 1×10^5^ of either CD271^+^, CD133^−^, CD133^+^ or unsorted cells for CD133 (upper row) and HMB45 (center row), respectively. A representative tumor out of n = 3 is shown. H&E shows histological morphology of tumors (lower row). Haematoxylin served as counter stain for HMB45. Bar size is 50 µm. (**C**) Immunofluorescence analysis for CD271 and CD133 on sections of tumors described in (A) showing co-localization and discrete expression of both markers. Scale bars indicate 50 µm. Nuclei were stained with DAPI (blue). (**D**) Growth of tumors following injection of 1×10^5^ of either CD271^+^, CD133^−^, CD133^+^ or unsorted cells was monitored for 57 days. Tumor volumes are shown as mean values ± SD of biological triplicates. Growth of 10^6^ of CD271^+^ cells is not shown. (**E**) Comparison of mRNA expression levels of either CD271^+^ cells with respective xenograft tumors derived from injection of CD271^+^ cells at different cell numbers (10^5^, 10^6^; left chart) or comparison of CD133^+^ cells with tumors as pointed (right chart). Expression in CD133^+^ cells was compared to xenograft tumors shown in (B) derived from injection of 10^5^ of CD133^−^, CD133^+^ or unsorted cells. mRNA expression levels of CD271, CD133, SOX10, MITF, MART-1 and TYR were determined by qPCR as indicated. Expression levels reveal the independence of the *in vivo* differentiation capacity of melanoma cells from the initial cell number or the cells phenotype. Shown are ΔCT values normalized to β-actin as mean value ± SD of biological triplicates. *p≤0.05; **p≤0.01 (t-test).

Our gene expression data albeit on transcriptional level, suggest a connection of CD271 and CD133. To figure out a potential relationship of both markers we have analyzed the cell surface expression of CD271 and CD133 of 10 patient-specific melanoma metastases-derived cell strains as well as cell lines MeWo and A375 by flow cytometry (Figure S3A–E). CD271^+^ cells were observed in 6/10 patient-derived cell cultures, in A375 and MeWo cells whereas a strong expression of CD133 was observed in just 3/12 cell strains. Among these cell strains we observed a strong variety in the number of cells expressing CD271 or CD133, respectively. As cells positive for CD271 or CD133 have been reported to be the tumor-initiating fraction of melanoma we asked if these sub-populations differ in terms of their capacity to form differentiated tumors. In order to determine the tumorigenic potential of both cellular sub-sets we FACS-isolated cells with a high expression of CD271 (Figure 2A) or positive or negative for CD133 (Figure S3F) from cell cultures, respectively. Then, up to 1×10^5^ cells sorted or unsorted for CD133 and different cell numbers of CD271^+^ cells (10^5^,10^6^) were injected into NSG mice. Fifty-seven days after injection all cell populations gave rise to tumors expressing the melanoma marker HMB45. We observed expression of CD133 in all tumors irrespective of the initial enrichment of injected cells for CD271 or CD133 expression (Figure 2B). Within the areas of CD271^+^ or CD133^+^ expressing cells we also observed a sub-set of CD271^+^/CD133^+^ positive cells (Figure 2C). Following injection of 10^5^ cells of each fraction we found no significant differences in tumor volumes of either CD133^−^ and CD133^+^, unsorted or CD271^+^ cells, respectively, suggesting a similar tumorigenic capacity of all cellular fractions (Figure 2D and Table S2). By quantitative RT-PCR of differentiation markers MITF-M, MART-1 or TYR we confirmed the *in vivo* differentiation potential of cell fractions enriched for either CD271 or CD133, respectively. The expression of differentiation markers was only marginally dependent from the number of injected cells (Figure 2E). qPCR further revealed increased levels of SOX10 in developed tumors compared to levels in CD271^+^ cells prior injection. Compared to CD133^+^ cells, qPCR showed lower basal levels of differentiation markers *MART-1* and *MITF-M* in CD271^+^ cells.

### The label-retaining compartment harbors cells with different phenotypes

**Figure 3:**
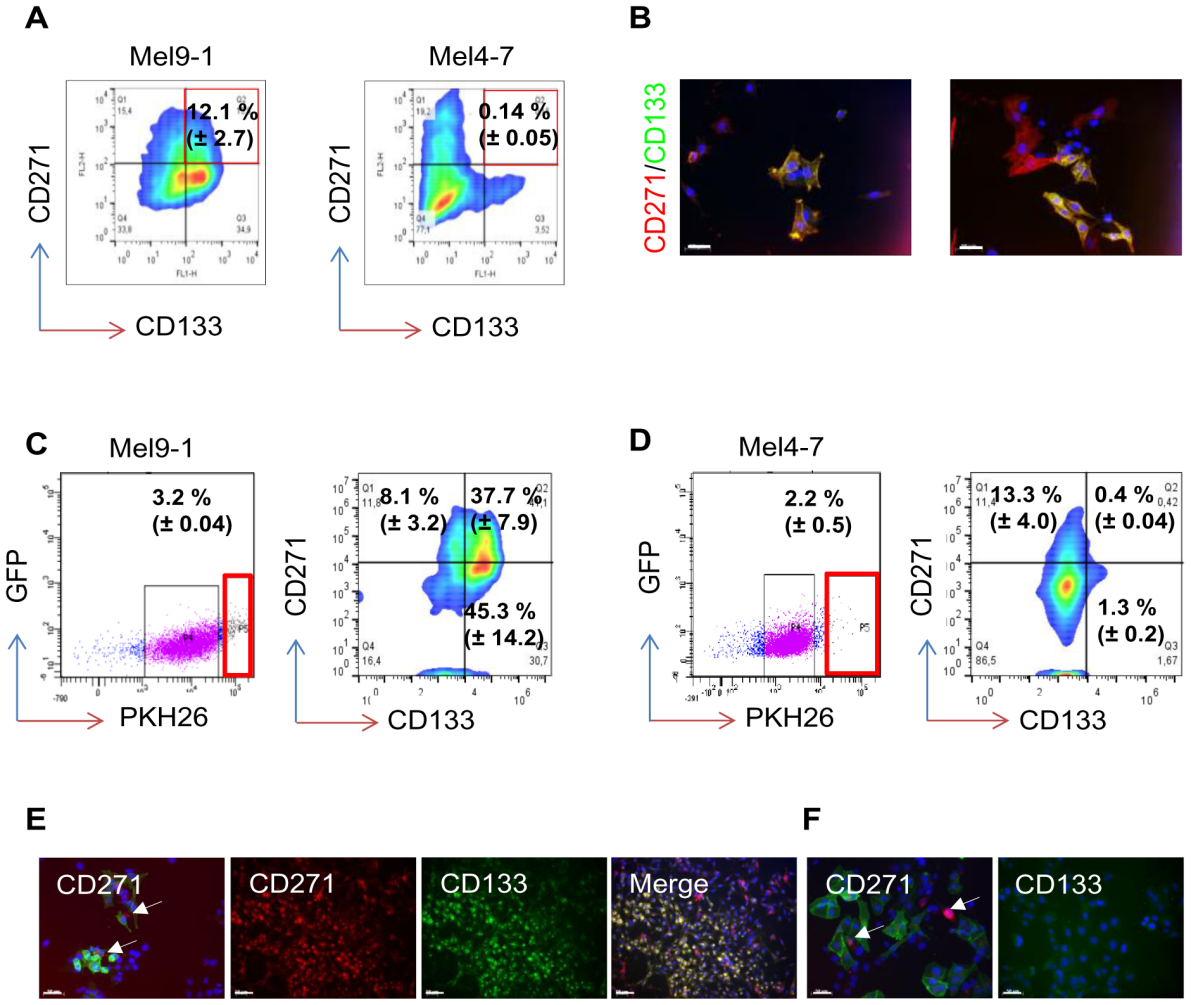
The label-retaining melanoma cell-fraction comprises CD271/CD133 double positive cells. (**A**) Co-expression of CD271 and CD133 in melanoma cell cultures shown by flow cytometry. The double positive fractions are highlighted and indicated as % ± SD of biological triplicates. (**B**) Immunofluorescence microscopy for co-expression of CD271 and CD133 in cell cultures shows a discrete or simultaneous expression of these markers. Scale bars indicate 50 µm. Nuclei were stained with DAPI (blue). Representatives of n = 5 are shown. (**C–D**) Isolation of highly fluorescent cells that retained the lipophilic dye PKH26 of two melanoma cell strains (Mel9-1 and Mel4-7, left panels) by FACS, 7 days after labeling. PKH26^high^ fractions are indicated as % ± SD of n = 3 independent FACS experiments. Confirmation of isolated, dye retaining fractions for presence of CD271^+^ and CD133^+^ cells by co-labeling and flow cytometry (right panels). Analysis shows the presence of cells with discrete and co-expression of markers. Representative plots indicate fractions as % ± SD of n = 3 independent experiments. (**E**) Analysis of isolated dye-retaining cells 7 days after sorting supports the co-localization of CD271 (green) and PKH26 (red, white arrows, first panel) or co-expression of CD271 (red) and CD133 (green) (panels 2–4). (**F**) Co-localization of CD271 (green) and PKH26 (red, white arrows, first panel) and low expression of CD133 in Mel4-7 cells. Scale bars indicate 50 µm. Nuclei were stained with DAPI (blue). Representatives of n = 3 are shown.

To find out if CD271^+^/CD133^+^ cells can be maintained in cell culture, we analyzed low passage bulk cell cultures for co-expression of both markers by flow cytometry. We found a relatively high amount (12.1%±2.7 and 0.14%±0.05) of CD271^+^/CD133^+^ cells in cell cultures of Mel9-1 and Mel4-7, respectively (Figure 3A). Immunofluorescence analysis showed co-localization as well as discrete localization of CD271 and CD133 (Figure 3B and S4A).

Asymmetric cell division and differences in doubling times may commonly guide to cellular heterogeneity. As the ratio of CD271^+^ and CD133^+^ cell populations remained stable we measured the retention of the fluorescent lipophilic dye PKH26, which should identify slowly dividing cells. The majority of initially labeled cells lost the dye within 5–7 days. However, 7 days post labeling flow cytometric analysis revealed a small sub-set of label-retaining cells that we subsequently isolated by FACS and analyzed the expression of CD271 and CD133 of recovered cells (Figure 3C). We observed label-retention for up to 14 days after sorting (Figure S4B–C). Dye-retaining Mel9-1 cells had either a CD271^+^/CD133^+^ (37.7%±7.9) or CD133^+^/CD271^−^ (45.3%±14.2) phenotype and were observed at lower frequency (8.1%±3.2) with a CD271^+^/CD133^−^ phenotype (Figure 3C). In a second cell strain with low expression of CD133, the majority of dye-retaining Mel4–7 cells was CD271 positive (13.3%±4.0) and only a minor fraction was double positive (0.4%±0.04) or positive for CD133 (1.3%±0.2) (Figure 3D). However, we also observed negative cells in the label-retaining fractions of both cell strains as compared to isotype control (Figure S4D). Analysis of Mel9-1 cells with a lower retaining capacity (shown in purple) revealed a reduced expression of CD271 (0.61%, data not shown) and increased fraction of negative cells (60.2%, data not shown). Mel4–7 cells with a lower retaining capacity have not been analyzed. Immunofluorescence analysis of dye-retaining Mel9-1 cells grown for 7 days after sorting confirmed the robust expression of CD271 and CD133 (Figure 3E), whereas the majority of dye-retaining Mel4–7 cells was CD271^+^ but extremely rare for expression of CD133 (Figure 3F). The isolation of cells in three phenotypes CD271^+^/CD133^−^, CD133^+^/CD271^−^ and CD271^+^/CD133^+^ by dye retention suggests these cells as part of the slow-dividing melanoma cell fraction.

### CD133^+^ melanoma cell cultures comprise both asymmetrically and symmetrically dividing cells

**Figure 4:**
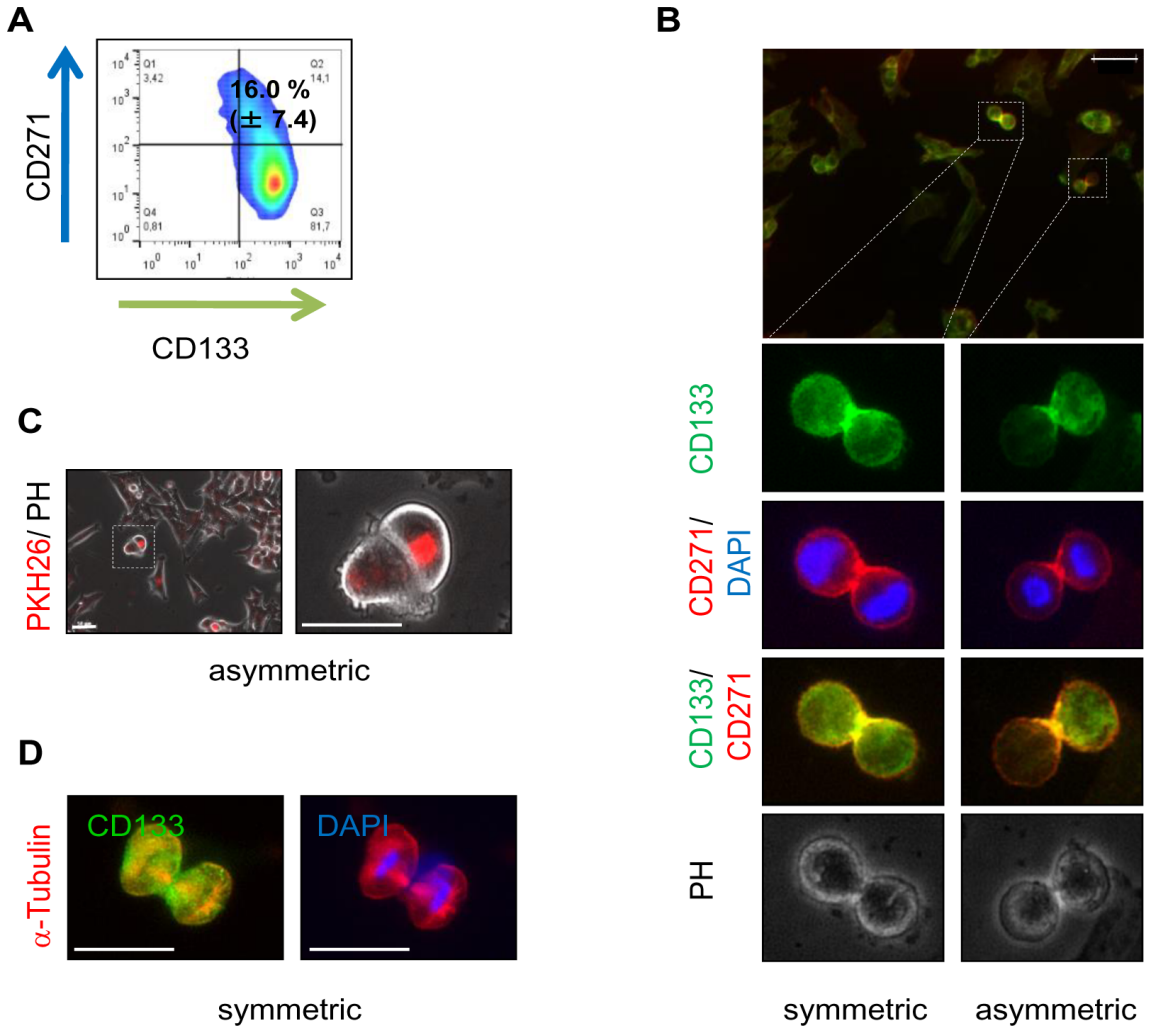
Asymmetrically dividing cells are CD133^+^ or double positive. (**A**) Flow cytometry for CD271 and CD133 in CD133^+^ enriched cells. Results depict the presence of single and double positive cells. The experiment was carried out in independent biological triplicates, a representative example is shown. (**B**) Distribution of CD271 and CD133 in symmetrically and asymmetrically dividing CD133^+^ cells detected by immunofluorescence microscopy for both markers. (**C**) Asymmetric cell division in unsorted cells is illustrated by the asymmetrical retention of PKH26. (**D**) Symmetric cell division is demonstrated by the symmetric orientation of microtubules indicated by α-Tubulin staining (red) and symmetrical distribution of CD133 (green). Scale bars indicate 50 µm. Nuclei were stained with DAPI (blue).

Considering our previous findings, we next analyzed the mode of cell division by immunofluorescence microscopy. As CD133 expression was linked with asymmetrical division of glioma cells [19], we expected the number of asymmetrically dividing melanoma cells to be highest in CD133^+^ enriched or double positive cells. In CD133^+^ enriched cells we observed a sub-set of CD271^+^/CD133^+^ (16.0%±7.4) (Figure 4A). We next investigated the distribution of CD271 and CD133 in dividing cells of the CD133^+^ fraction and observed the asymmetrical distribution of CD133 but not CD271 predominantly in CD133^+^ cells (Figure 4B). In the CD133^+^ fraction, asymmetric cell division, although rare, may be responsible for the observed cellular heterogeneity. Cells that underwent asymmetrical cell division were further evident in a minority of unsorted cells as indicated by label retention (Figure 4C). However, the vast majority of unsorted cells underwent symmetric cell division (Figure 4D).

### Development of CD133^+^ cells *in vitro* depends on growth factor conditions

**Figure 5:**
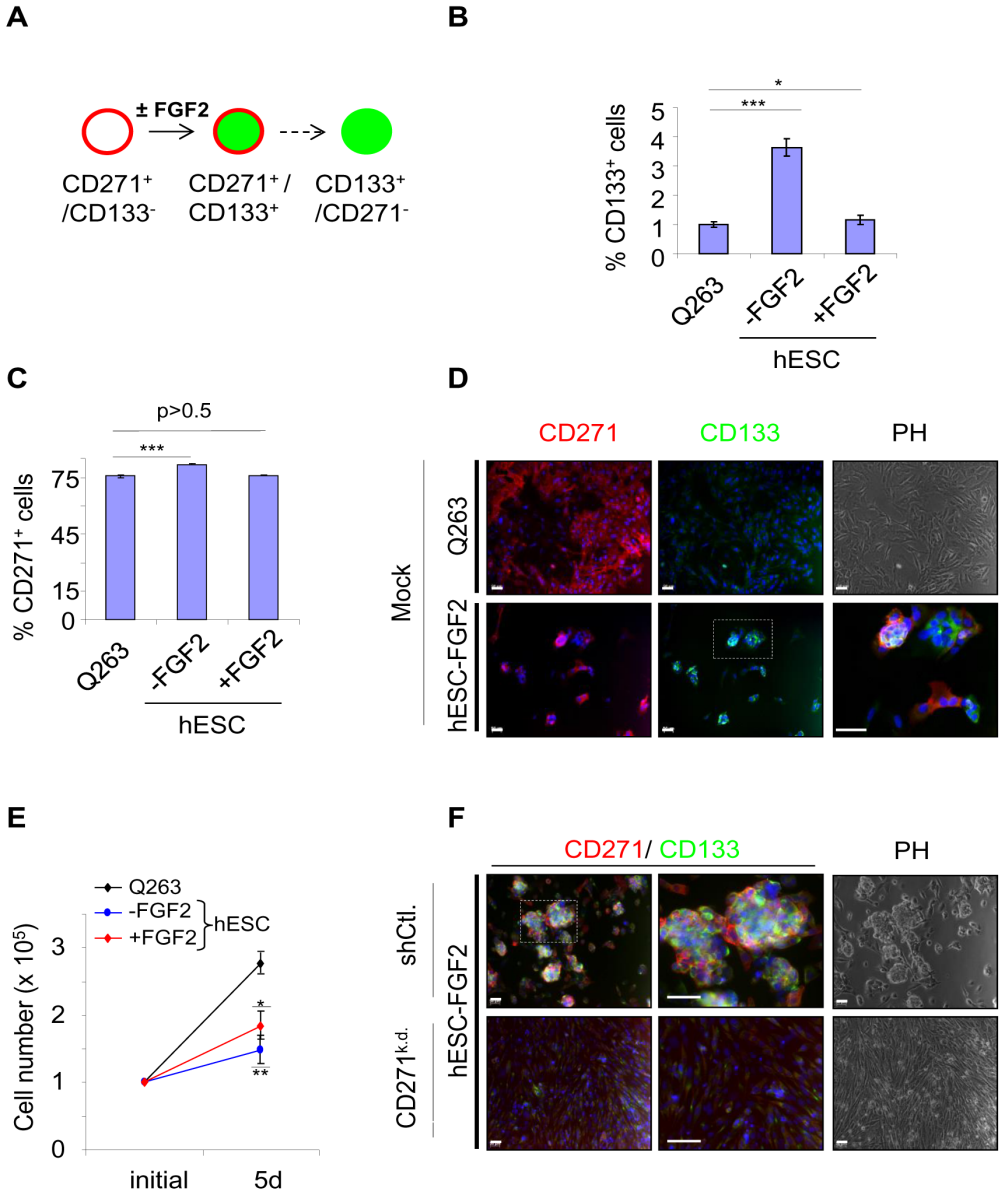
Melanoma cells can adopt a CD133^+^ phenotype by changes of extracellular cues. (**A**) Proposed model of phenotypical transitions of melanoma cells in dependence of FGF2. In this model, a CD271^+^/CD133^−^ cell could become CD271^+^/CD133^+^. In a second step, this cell may convert into a CD271^−^/CD133^+^ cell. (**B–C**) Unsorted melanoma cells were grown in standard (Q263) or human embryonic stem cell (hESC)-medium in absence or presence of FGF2 for 5 days. The amount of CD271 and CD133 positive cells was determined by flow cytometry. Bar graphs indicate the amount of CD133^+^ or CD271^+^ fractions in % ± SD of biological triplicates. (**D**) Immunofluorescence microscopy of untransfected (Mock) cells cultured either in Q263 (upper row) or hESC-medium in absence of FGF2 (lower row) for CD271 (red) and CD133 (green). Double positive colonies are shown in the bordered area and in the magnification. Scale bars indicate 50 µm. (**E**) Growth of melanoma cells is shown as cell counts ± SD of n = 3 experiments. 1×10^5^ cells were plated initially and cultured for 5 days under different conditions as indicated. *p≤0.05; **p≤0.01 (t-test). (**F**) Immunofluorescence microscopy of shCtl. and CD271^k.d.^ cells grown under colony-forming conditions indicating the formation of CD271^+^/CD133^+^ colonies by shCtl. cells but not CD271^k.d.^ cells. Phase contrast (PH) depicts cellular morphology. Scale bars indicate 50 µm. Nuclei were stained with DAPI (blue). Representatives of biological triplicates are shown.

CD133 and CD271 double positive cells were frequently observed next to CD271 single positive cells in xenograft tumors. Therefore, we asked whether these cells could develop from CD271^+^ cells and if they are capable of generating cellular heterogeneity *in vitro*. Cells were cultivated in hESC-medium in presence or absence of fibroblast growth factor 2 (FGF2). Cultivation of cells in absence of FGF2 for five days changed the ratio between CD271 and CD133 expressing populations (Figure 5A). Flow cytometry revealed an increase of 3.6% (±0.3) CD133^+^ cells in hESC-medium without FGF2 compared to supplemented media (Figure 5B). In addition, the relative amount of CD271-expressing cells was slightly increased in absence of FGF2, but remained unchanged in its presence (Figure 5C). Cells cultivated in hESC-medium in absence of FGF2 formed round-shaped colonies within 2 days and were growth deficient. Proliferation of cells was only marginally increased by FGF2. Subsequently colonies displayed high expression of CD133 and CD271 (Figure 5D–E).

### The formation of CD133 positive cells *in vitro* is associated with expression of CD271

To elucidate if the formation of CD133 expressing cells depends on the presence of CD271, we analyzed the effect of shRNA mediated CD271 knock-down on the ability to (re)gain CD133 expression in the absence of FGF2. We found that the competence of cells to form CD133^+^ colonies is lost upon knock-down of CD271 but was not affected in shCtl. cells. The latter one displayed a comparable colony forming capacity to Mock cells (Figure 5F). To find out further, if CD271^+^ cells could spontaneously convert into CD133^+^ cells, we enriched CD271^+^/CD133^−^ Mel9-1 cells to a yield of 72.7% (Figure S5A). After 3 days of cultivation we performed immunofluorescence microscopy (Figure S5B) and calculated the ratios for CD271 (85%) and CD133 (34.2%±5.9) cells (Figure S5C). The majority of CD133^+^ cells was also positive for CD271 (CD271^+^/CD133^+^).

As we suppose the derivation of CD133^+^/CD271^−^ cells from a CD271/CD133 double positive population, we have analyzed double positive sorted cells 3, 5 and 7 days after isolation (enriched to 93.1% purity; data not shown) for the ratios of CD271^+^/CD133^+^ and CD271^−^/CD133^+^ cells, respectively. Although the CD271^+^/CD133^+^ sub-population remained stable for 3 days (90.0%±0.8) we did observe a sub-population of CD133^+^ cells (6.8%±0.6) that significantly increased after 5 days (17.2%±1.6) and 7 days (26.2%±0.9), (Figure S5D–E). Sorted cells were subjected to PKH26-labeling. The vast majority of cells retained the dye after 3 days of cultivation but only a small fraction was highly label retaining after 7 days (Figure S5F). We verified these findings independently with Mel4-7 cells by confirming that cells sorted for either CD271^+^/CD133^−^ or absence of both markers (CD271^−^/CD133^−^) gave rise to CD271^+^, CD271^+^/CD133^+^ and CD133^+^ cells (Figure S6A–B). Sorted cells were clearly morphologically distinct from unsorted cells with an epithelial-like and cluster-forming morphology of CD271^+^/CD133^−^ cells and a spindle-like morphology of CD271^−^/CD133^−^ cells (Figure S6C). The conversion of CD271^+^/CD133^−^ sorted cells towards cells showing a distinct or double expression of these markers was further verified by immunofluorescence microscopy (Figure S6D). These data strongly suggest CD271 as to be responsible for colony-formation and the association of this molecule with melanoma cell plasticity.

To elucidate a potential mechanism which may trigger the loss of CD271 expression hence the phenotypic conversion of cells from CD271^+^/CD133^−^ to CD133^+^/CD271^−^, we tested the effect of TGFβ2 on shCtl. cells. TGFβ is known to epigenetically modify the expression of CD133 and reduce the level of SOX10 during the derivation of mesenchymal progenitors from neural crest stem cells [20]. While CD133 expression was not induced (Figure S6E), we observed activation of the TGFβ-signaling pathway as reasoned by an increased frequency of cells expressing pSmad2 after treatment of cells for 3 days while a strong reduction of SOX10 expression was confirmed by immunofluorescence microscopy.

### CD271^+^ cells show an undifferentiated phenotype

**Figure 6:**
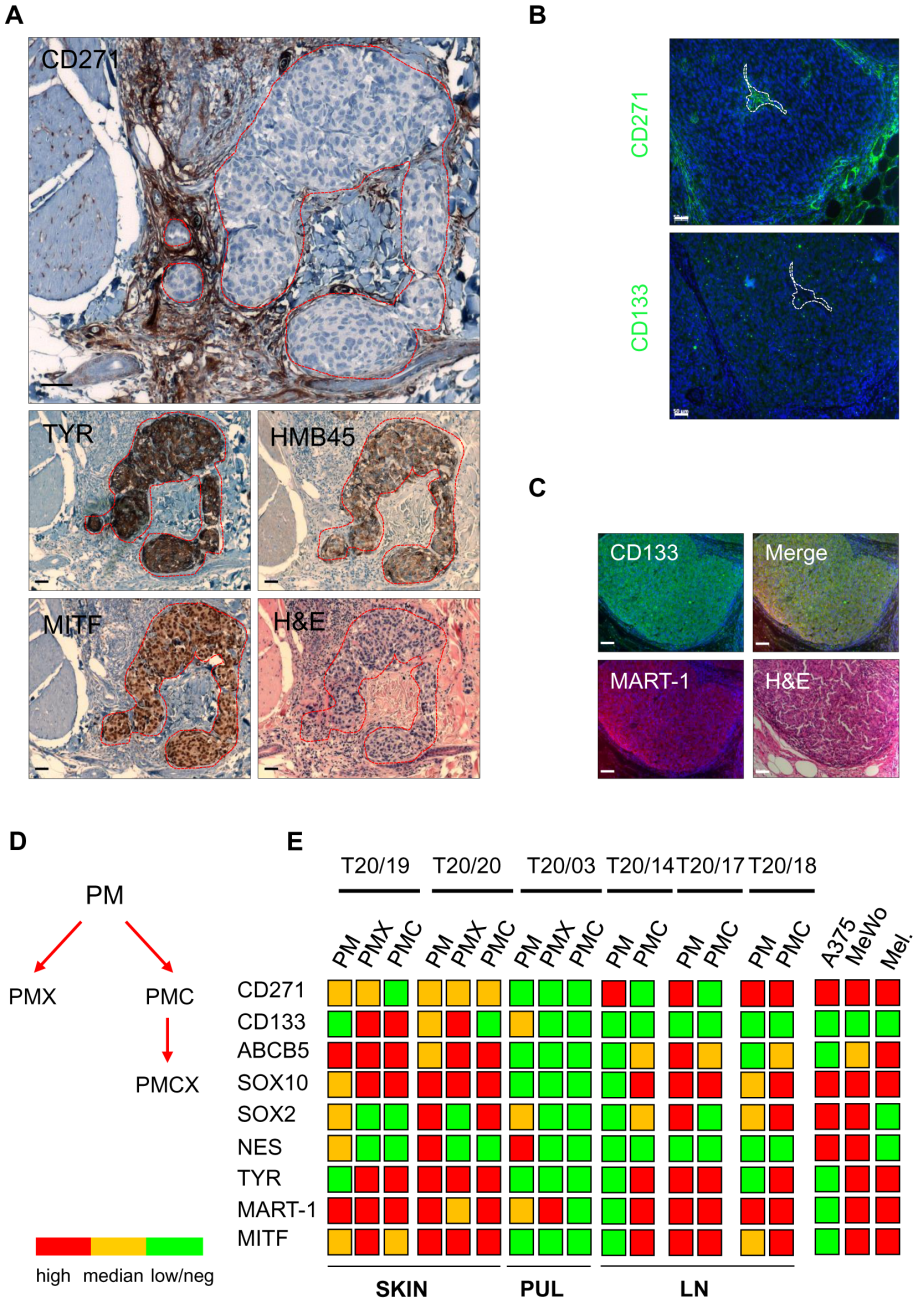
CD271 is a predominant marker of malignant melanoma. (**A**) Immunohistochemistry (IHC) of CD271 and melanoma differentiation markers TYR, HMB45 and MITF of a representative melanoma metastasis (skin). The mutually exclusive expression of CD271 is marked by the red dashed line. Haematoxylin was used as a counter stain in IHC and H&E discriminates differentiation marker positive cells with large nuclei from negative cells with small nuclei. Scale bars indicate 50 µm. (**B**) Immunofluorescence microscopy of serial sections of a hepatic metastasis (patient T20/15) shows expression of CD271 and absence of CD133. (**C**) Co-expression and membranous localization of CD133 and the melanoma antigen MART-1 of a representative out of 16 tumors. Scale bars indicate 50 µm. Nuclei were stained with DAPI (blue). (**D**) Illustration of patient-derived melanoma metastases (PM) derivatives: PMX, PM derived xenografted tumors; PMC, PM derived established cell strains; PMCX, PMC derived xenograft tumors. (**E**) Roundup of qPCR results of PM, PMX and PMC. Shown are the expression levels of *CD271*, *CD133*, *ABCB5*, *SOX10*, *SOX2*, *NES*, *TYR*, *MART-1* and *MITF* (*MITF-M*) (n = 6). The color code indicates high (red) or median (yellow) expression or low/absence (green). A375, MeWo and human melanocytes served as controls (metastases of SKIN  =  cutaneous; PUL  =  pulmonary/lung and LN  =  lymph node).

Our *in vitro* data demonstrate a predominance of CD271 but a minor appearance of CD133 expressing cells. To dissect whether the observed distribution of both cellular sub-sets was due cell culture conditions that selected for robust cell types exhibiting higher survival and proliferative capacity or can also be found in patient-derived tumors, we have investigated the distribution of CD271 and CD133 positive cells in 16 patient-derived metastases. We found that CD271 was expressed in all tumors investigated, independent of the origin of the primary tumor and the location of the respective metastasis (Figure S7A). Areas positive for CD271 were shaped nodule-like with clear boundaries. We did not observe infiltration into the surrounding tissue. Expression of CD271 and of the differentiation markers TYR, HMB45 and MITF was mutually exclusive (Figure 6A and Figure S7B–C). We did not observe a co-expression of CD271 and CD133 in these tumors (Figure 6B) as expression of CD133 was limited to regions positive for the differentiation marker MART-1 (Figure 6C). These observations support our *in vitro* data and the finding of a CD271-dependent stem-like state found by GSEA thus suggest CD271 as a predominant molecule in malignant melanoma.

### Heterogeneity among melanoma metastases is conserved in xenograft tumors

We further investigated the heterogeneity of patient-derived metastases (PM), PM-derived xenograft tumors (PMX) and *in vitro* established PM-derived cell strains (PMC) as well as a PMC-derived xenograft tumor, respectively (Figure 6D) by qPCR. Analysis of mRNA expression levels of CD271, CD133, *ABCB5*, *SOX2* and melanoma markers *SOX10*, *NES* and *TYR*, MART-1 and *MITF*-M revealed a strong heterogeneity among the PM tissue samples (Figure 6E). However, we observed comparable expression levels between patient-derived metastases and their respective xenograft tumors in 3 patients (T20/19; T20/03; T20/18). By qPCR, we found CD271 expressed in all tumors investigated albeit in one patient only at low level (T20/03). Immunohistochemistry for CD271 and MITF of PM and PMX of patients T20/19 and T20/20 demonstrated comparable heterogenic patterns of expression (Figure S8A). Additionally, xenograft tumors of unsorted cultured cells (PMCX) of patient T20/02 revealed a comparable heterogeneity in terms of CD271 and MITF expression as found in the primary metastasis (Figure S8C). Even though we observed in part strong variations of marker gene expression of metastases-derived cell strains established from patients' tumors (T20/03, T20/14 and T20/17–20) in comparison to PM. However, expression patterns found in PMC were comparable to established cell lines A375 and MeWo. These data demonstrate the persistence of CD271^+^ cells in metastases-derived xenograft tumors as well as in tumors of cultured cells.

## Discussion

The fact that melanocytes or melanoma cells express genes that can be similarly found in their developmental ancestor, the neural crest stem cell (NCSC), may implicate commonalities in the regulation of cellular properties.

**Figure 7:**
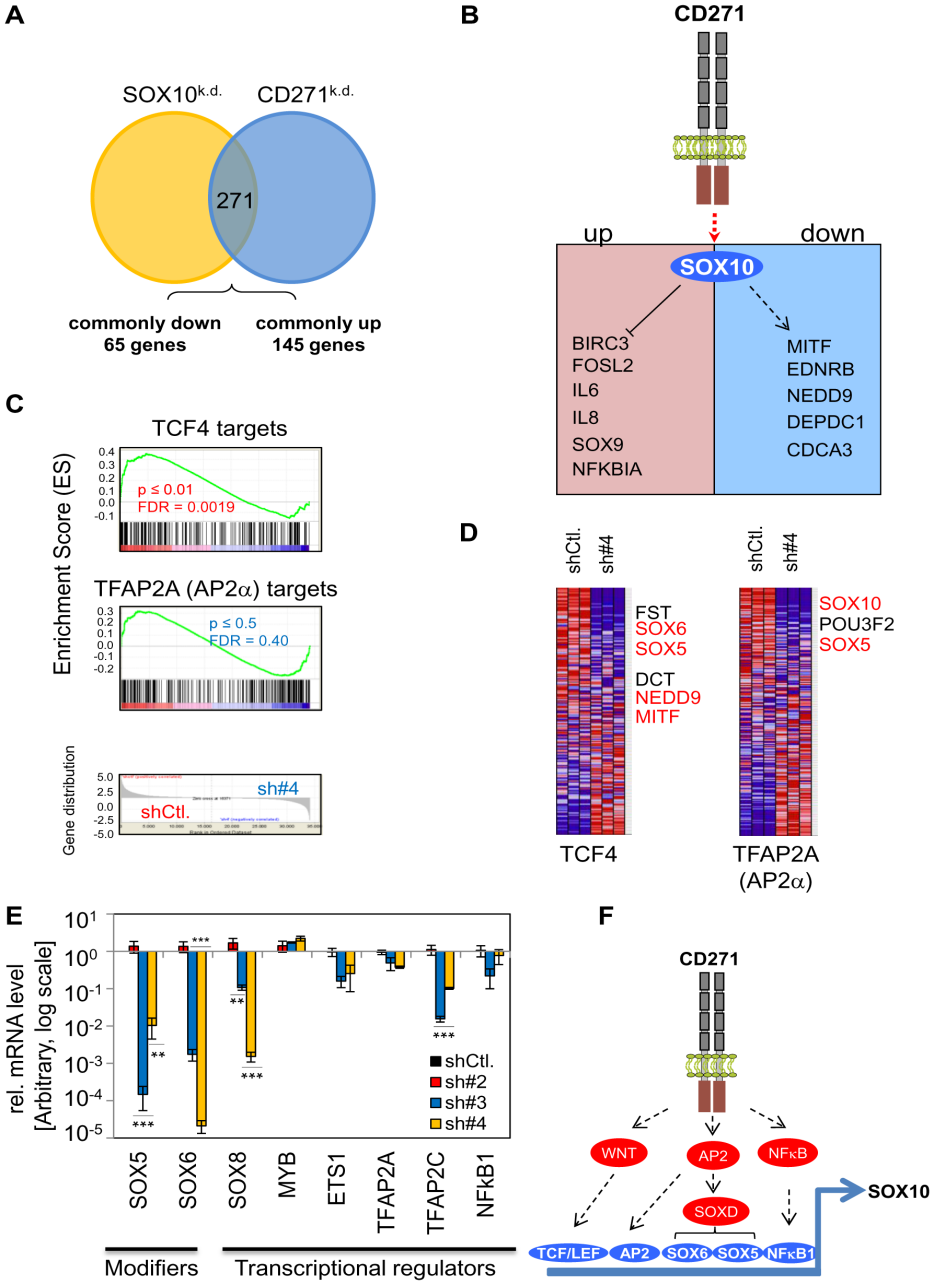
CD271 and SOX10 are main regulators of a melanoma-associated signaling network. (**A**) Venn-diagram depicting the meta-analysis of genes differentially regulated in shCtl. vs. CD271^k.d.^ cells (green) and a data set of shCtl. vs. SOX10^k.d.^ cells (red) [9]. The analysis revealed 271 shared regulated genes among them 65 were found as commonly down-regulated (ratio <0.75) and 145 as commonly up-regulated (ratio >1.33). (**B**) Model of a yet unknown signaling mechanism that leads to activation of a SOX10-dependent network triggered by CD271. The scheme depicts several genes we found regulated by CD271/SOX10 in (A). Up-regulated genes were representatives of the NFκB family e.g. baculoviral IAP repeat containing 3 (*BIRC3*), FOS-Like antigen 2 (*FOSL2*), interleukins 6 and 8 (*IL6/8*), SRY-box transcription factor 9 (*SOX9*) and the nuclear factor of kappa light polypeptide gene enhancer in B-cells inhibitor, alpha (*NFKBIA*). Down-regulated genes were the microphthalmia-associated transcription factor (*MITF*), endothelin receptor b (*EDNRB*), neural precursor cell expressed, developmentally down-regulated 9 (*NEDD9*), DEP domain containing 1 (*DEPDC1*) and cell division cycle associated 3 (*CDCA3*). (**C–D**) Comparison of data sets of shCtl. and CD271^k.d.^ cells with data sets of either a whole-in situ hybridization study in *Xenopus* [51] or a RNAse-protection screen for AP2α binding sites [52] by gene-set enrichment analysis (GSEA). GSEA revealed the presence of TCF4 and TFAP2A (AP2α) regulated genes in shCtl. cells. The TCF4 and TFAP2A signatures are lost upon CD271 silencing. Beside known WNT/TCF4 target genes *MITF*, *DCT* and *FST* (Follistatin), *SOX5*, *SOX6* and *NEDD9* were identified (first panels). GSEA for putative AP2α regulated genes revealed *SOX10*, *POU3F2* and *SOX5* enriched in shCtl. cells. (**E**) Analysis of mRNA expression levels of modifiers *SOX5* and *SOX6* and inducers *SOX8*, *MYB*, *ETS1*, *TFAP2A*, *TFAP2C* and *NFκB1* of *SOX10* expression by qPCR. Expression levels of shRNA control (shCtl.) cells and CD271^k.d.^ cells are shown as ΔΔCT values normalized to β-actin and related to shCtl. cells as mean value ± SD of biological triplicates. **p≤0.01; ***p≤0.001 (t-test). The scale is logarithmic (log). (**F**) Scheme of putative CD271-dependent signaling pathways. GSEA and qPCR may point out a connection of CD271 and a WNT-dependent regulation of TCF/LEF target genes, activation of AP2-dependent target genes like SOX5 that in turn regulates SOX6 or activation of NFκB signaling.

Here, we deciphered novel connections of CD271 with known melanoma-associated genes and demonstrated that expression of CD271 determines specific properties of melanoma cells and melanoma-initiating cells like proliferation, cellular heterogeneity and tumorigenicity. We found, that CD271 knock-down abolished the capacity of melanoma cells to form heterogeneous tumors most likely due to down-regulation of mediators of melanoma invasion and metastasis (*GLI-2* [21], *SOX2* [22] *and ERBB3* [23]), angiogenesis (*IGFBP-2* [24]), proliferation (*FST* [25] and *MITF*) or chemoresistence (*RHOJ* [26]). In addition, GSEA revealed that some of these genes are crucial for NCSC maintenance and migration, like *SOX10* and *SNAI2* or *SEMA3C* [27], respectively. We found *SOX10* as one of the most down-regulated genes in CD271^k.d.^ cells. As both CD271^k.d.^ cells and SOX10^k.d.^ cells that were analyzed prior by Shakova et al. [9] were unable to induce tumor formation in a mouse model system, we strongly suggest that CD271 acts in concert with or via SOX10 to regulate melanoma cell properties e.g. tumorigencity. This idea is underpinned by the finding of a set of 271 overlapping genes including 65 genes as commonly down-regulated and 145 genes as commonly up-regulated, respectively (Figure 7A). Among the down-regulated genes, we found *MITF* which controls proliferation and invasiveness of melanoma cells [28] and the endothelin receptor b (*EDNRB*) which is strongly associated with melanoma development [29] and central nervous system directed metastasis [30]. We also found the *bona fide* driver of melanoma metastasis and invasion *NEDD9* [31] as well as *DEPDC1* and *CDCA3*, genes involved in progression of bladder cancer [32] or enhanced proliferation [29]. Additionally, we found mediators of the NFκB pathway among those repressed by SOX10 and CD271 (Figure 7B). Among the commonly regulated genes our meta-analysis identified 52 independently regulated genes and 60 reverse regulated genes like *NAV3* or *MSH5* (data not shown). However, genes belonging to either of the groups have not been analyzed in more detail, yet. Although CD271 as a member of the TNF-receptor family should lead to activation of the NFκB pathway this pathway seems to be inactive in CD271^+^ melanoma cells and is activated when the receptor is lost.

SOX10 is known to act as a specifier of neural crest derivatives [30], [33] and directly regulates melanoma associated genes like *ERBB3* [34], *EDNRB* [35] and *MITF* [36]. Further, CD271 expression was reduced in SOX10^k.d.^ cells, suggesting a mutual yet unknown regulation. Our findings confirm previous observations regarding the role of CD271 and SOX10 in melanoma cells [14], [15]. However, within these studies a connection of CD271 with other melanoma-associated genes has not been analyzed. To gain further insight into a potential regulatory network connecting CD271 and SOX10 we performed GSEA and qPCR analysis. We confirmed potential transcriptional regulators of SOX10 expression like SOX8, MYB, ETS1, AP2 (TFAP2A and TFAP2C) identified previously by Betancur et al. as well as mediators of WNT and NFκB signaling like TCF4 and NFκB1, respectively. Although binding of SOXD proteins SOX5 and SOX6 was found by *in silico* analyses only, the significance of especially SOX8, MYB and ETS1 for SOX10 expression has been clearly demonstrated in chicken embryos [37]. In addition, the expression of SOX10 was shown to rely on canonical WNT signaling in *Xenopus* embryos [33] and is modified by SOX5 in a complex fashion [38]. By GSEA we found genes regulated in a TCF4-dependent manner lost in the CD271^k.d.^ cells (Figure 7C). Among known targets of WNT-signaling like MITF [39] and Follistatin (FST) [40], we found NEDD9 a gene we observed previously by meta-analysis and SOX5 and SOX6 (Figure 7D). SOX10, Brn2 (POU3F2) and SOX5 among others were identified in the group of TFAP2A target genes. However, we did not observe a clear discrimination of genes enriched in shCtl. cells and those enriched in CD271^k.d.^ cells, suggesting a minor role of TFAP2A in the CD271-dependent regulation of SOX10. Finally, we have analyzed expression levels of putative modifiers and inducers of SOX10 expression by qPCR and found a strong and significant down-regulation of SOX5, SOX6, SOX8 and TFAP2C (AP2γ), a moderate down-regulation of ETS1, TFAP2A (AP2α) and NFκB1 as well as a slight up-regulation (∼2fold) of MYB (Figure 7E). In summary, the regulation of SOX10 expression in melanoma is supposedly comparable with the mechanism that controls SOX10 during development and specification of the neural crest. Therefore, CD271 may be involved in the regulation of different pathways like WNT, NFκB and activation of AP2 (Figure 7F) controlling SOX10 in a direct or indirect fashion. Although we are unable to dissect the connection of CD271 and SOX10, yet our study provides a deeper insight in the CD271-dependent signaling network and reveals that this network comprises several genes known as drivers of melanoma progression and metastasis.

Besides the direct correlation of CD271 and SOX10, we found in our present study, that expression of CD133, at least in part, was dependent from the level of CD271. We observed CD133 up-regulated in CD271^k.d.^ cells of a patient-derived melanoma cell strain by genome-wide expression profiling and qPCR. In opposition, the over representation in CD271^exo^ cells led to down-regulation of CD133. As we could confirm the correlation of CD271 and SOX10 or CD133 in MeWo cells a regulation of both markers by a CD271-dependent signaling mechanism seems very likely. However, increased cell surface levels of CD133 were not observed in CD271^k.d.^ cells.

Changes in gene expression upon down-regulation of CD271 not only affect melanoma cell properties but may also induce a cell-lineage switch from neural-like to mesenchymal-like. Interestingly, we found TGFβ-induced genes enriched in CD271^k.d.^ cells by GSEA (Figure S9A). TGFβ-signaling is known to suppress SOX10 expression and was shown to induce CD133 expression by down-regulation of DNA-methyltransferases 1 and 3 b and a concurrent de-methylation of the CD133 promoter [41], [42]. Therefore, the acquisition of a mesenchymal-like phenotype and increased TGFβ-signaling may also be responsible for both the down-regulation of SOX10 and the increase of CD133 mRNA level. The mutually exclusive expression of SOX10 and CD166 we observed in MeWo cells upon silencing of CD271 may underpin this hypothesis.

Further we observed that tumorigenic CD271^+^ cells capable of differentiation were maintained in bulk melanoma cell cultures and were also maintained by the microenvironment of the xenograft tumor. This “robustness” might indicate the importance of CD271^+^ cells for maintaining a viable population. In contrast, the low occurrence of cell strains comprising CD133^+^ cells may be due to a loss upon cell culturing. This loss could be explained by a decreased differentiation or regeneration of CD133 expressing cells or may be due to their sensitivity for the microenvironment. However, by xenotransplantation of CD271^+^ and CD133^+^ sorted cells we could rule out a rapid and intense differentiation of cultured CD133^+^ cells as demonstrated by their capability to form heterogenic tumors like their CD271^+^ counterpart. Hence, cells of both fractions fulfilled the criteria of tumor-initiating cells and evidenced their undifferentiated phenotype and suggest CD133^+^ and CD271^+^ being equally tumorigenic. The relatively high amount of CD133^+^ and CD271^+^ cells in unsorted cell populations seemed sufficient to allow tumor formation that was comparable to enriched cells. Nevertheless, the observed differences in levels of differentiation markers like HMB45, MART-1 and MITF-M in xenograft tumors of CD133^+^, CD133^−^ and CD271^+^ cells was due to different basal levels of these markers in cells prior to injection. In contrast to CD271^+^ cells, the MART-1 and MITF-M levels of CD133^+^ cells were indeed much higher as determined by qPCR. We speculate that CD133^+^ cells may generate a MART-1 expressing sub-population or turn into a CD133/MART-1 double positive population *in vitro* and, to a higher extend, *in vivo*. In addition, observations of CD271^+^/CD133^+^ cells in all tumors, irrespective of the initial cells phenotype and the development of CD133^+^ tumors from initially CD133^−^ sorted cells, strongly suggests the plasticity of melanoma cells. This plasticity is not necessarily caused by CD133 expression, but could be credited to the co-expression of CD271. We were unable to exclude that xenograft tumors developed from CD271 expressing cells rather than from cells exclusively expressing CD133. This is because in all experiments we detected cells that shifted from CD271^+^/CD133^−^ to the CD271^+^/CD133^+^ phenotype.

The amount of melanoma-initiating cells is expected to be low in the tumor due to a reduced proliferative capacity and the tendency to divide asymmetrically [43]. Thus these cells should be successively lost upon *in vitro* expansion. We demonstrated, at least in part, a high quantity of CD271^+^ cells in melanoma cell cultures, arguing against their role as melanoma-initiating cells. However, using dye retention assays we were able to identify slowly-dividing label-retaining cells as a minor fraction of melanoma cell cultures. Flow cytometry of isolated dye-retaining cells revealed their CD271^+^ phenotype found in independent experiments of two melanoma cell strains. This finding emphasizes the stem-like properties of at least a sub-set of CD271^+^ cells [11], [44]. In the Mel9-1 cell strain we observed the co-occurrence of CD271^+^, CD133^+^ and CD271^+^/CD133^+^ cells in the dye-retaining fraction suggesting the transition of CD271^+^/CD133^−^ into CD271^+^/CD133^+^ cells within this fraction. However, we also observed cells negative for both markers in the fraction of PKH26^high^ sorted Mel9-1 cells and to a higher extent in dye-retaining Mel4-7 cells. This and the observation of lower levels of CD271 and CD133 in cells with a lower retaining capacity suggest the dye-retaining fraction as being heterogeneous. Therefore label-retention is not directly linked to expression of CD271 and CD133 but enables the enrichment of melanoma cells for either of these markers.

Further we demonstrated that CD133^+^ cells developed from CD271^+^ cells in a growth factor reduced environment. With respect to offered growth factors we observed that the absence of FGF2 rather than its presence supported the cellular transition from CD271^+^/CD133^−^ to a CD271^+^/CD133^+^ phenotype. As this transition had also occurred spontaneously by CD271^+^/CD133^−^ cells that were isolated from a mixed cell culture comprised CD271^+^, CD133^+^ and CD271^+^/CD133^+^ cells, we assume a diverse role of FGF2. FGF2 may alter those signaling pathways that are already activated in a certain cell population by a specific mutation or by autocrine or paracrine mechanisms. In the tumor, FGF2 is secreted by stromal fibroblasts as part of the microenvironment [45], [46] and may serve either as a stem-cell maintaining or differentiating factor. As the standard medium, Q263, contained FGF2, we assume a repression of the transition towards a CD133^+^ phenotype that is activated in FGF2 free medium. Yet, we cannot ultimately decide if the cellular transition was the consequence of a stress-response triggered by a reduced growth factor environment or representative of a regenerative mechanism that “reactivates” dormant cells. The spontaneous transition of CD271^+^ sorted melanoma cells towards CD133^+^ in standard medium may also depend on the level of TGFβ-signaling and needs both low levels of CD271 and SOX10. We elucidated whether the level of SOX10 in melanoma cells changed by treatment with TGFβ2 and concomitantly led to expression of CD133. Indeed, the protein level of SOX10 strongly decreased by activated TGFβ-signaling but levels of CD133 remained unchanged. In addition, cultivation of cells in hESC-medium led to increased TGFβ-signaling indicated by pSmad2 irrespective whether FGF2 was present or absent (data not shown). Therefore, the identification of factors responsible for the transition of melanoma cells towards a CD133^+^ phenotype remain elusive and needs further investigations.

We assume the CD133^+^ phenotype as a marker of a pre-differentiated but tumorigenic melanoma cell that derived from a CD271^+^ cell as a function of microenvironmental changes. We further conclude that both cellular states, CD271^+^ and CD133^+^, are tightly controlled and well balanced. This statement is further underpinned by our observation that the amount of sorted CD271/CD133 double positive cells decreased in a time-dependent manner and led to derivation of CD133^+^/CD271^−^ cells due to a decreased level of CD271. Overexpression of CD271 or high endogenous levels may promote the stem-like state and may even keep melanoma cells from differentiation. Therefore a medium or low endogenous level of CD271 is needed for the transition towards the CD133^+^ state. CD271 silencing alleviates differentiation into a yet unknown cell type and may further sensitize cells towards drug-induced apoptosis by a reduced DNA-repair capacity and a decrease of anti-apoptotic genes as observed by immunofluorescence microscopy and qPCR, respectively. Therefore, further analyses are needed to fully understand how CD271 signaling can induce and maintain the stem-like state and determine melanoma cell properties.

Finally, we analyzed the distribution of CD271^+^ and CD133^+^ cells in patient-derived melanoma metastases and observed a predominance of CD271^+^ cells whereas expression of CD133 was less frequently observed. We investigated the expression of both markers in superficial spreading melanoma and acral lentiginious melanoma (SSM and ALM; types of cutaneous melanoma) and uveal melanomas and their respective metastatic sites. We found CD271 expression in all metastases investigated and respective xenograft tumors albeit with varying intensities and limited to tumor-initiating cells [47], negative for HMB45 and MITF. The mutually exclusive expression of CD271 and differentiation markers confirms previous findings by Boiko et al., though we have shown additionally that CD271^+^ cells persist in xenografted tumors and primary tumor derived cells strains and may contribute to the strong heterogeneity we observed among them by qPCR. The finding of CD133 co-expressed with MART-1 in primary metastases may support their more differentiated phenotype.

## Conclusions

We identified CD271 as a predominant molecule in malignant melanoma responsible for proliferation, tumorigenicity and plasticity of melanoma cells. The connection of CD271 with the transcription factor SOX10 points out a CD271/SOX10 network controlling these properties by yet unknown mechanisms. Additionally, melanoma cell plasticity linked CD271 to the glioma stem cell marker CD133 and clearly demonstrated a connection of both markers in malignant melanoma most likely via a CD271^+^/CD133^+^ intermediate cell state. However, melanoma cells and melanoma-initiating cells can be described by more than just a single marker. We cannot exclude that other cell surface markers like ABCB5 or transcription factors like SOX2 may participate or even act as critical factors in the transition of melanoma cells phenotype. Further, this transition may not only depend on secreted factors supported by the tumor microenvironment but may also be induced by chemotherapeutics. Taken together, these findings have strong implications not only for basic melanoma understanding, but potentially provide a laver to constrict tumor growth and metastasis.

## Materials and Methods

### Ethics Statement about Patient-derived material

Written informed consent was obtained from each patient. Patient's material was collected between 2010–2012 at the Comprehensive Cancer Center of the Charité University Hospital (CCCC) with coverage of the Tumor REsearch And Treatment – 20 Patient Pilot (TREAT 20) project approved by the Federal Ministry of Education and Research (No. 031 5852A). Tumor specimens were obtained at the time of surgery and spitted into equal pieces for paraffin-embedding and establishment of tumor-derived cell cultures. We received material sufficient for both procedures from 16 out of 20 patients.

### Cell culture

All primary low passage melanoma cell strains and A375 cells were cultured in Quantum 263 medium (PAA, Austria) supplemented with 1% Penicillin/Streptomycin (Invitrogen) while MeWo cells were cultured in Leibovitz's L-15 medium (Invitrogen) supplemented with 1% Penicillin-Streptomycin. Cells were cultured in 5% CO_2_, 37°C and routinely passaged at 80% confluence. Medium was changed every 3 days. Human neonatal epidermal Melanocytes were cultured in Melanocyte growth medium (both Lonza). Phenotype shift experiments were done in hESC-medium with and without 4 ng/ml of FGF-2 (Biosource) or in Quantum 263 medium supplemented for 3 days with 10 ng/ml TGFβ2 (Miltenyi).

### Magnetic Cell sorting (MACS)

MACS separation into CD133^+^ and CD133^−^ fractions was done with accordance to the manufacturer's instructions using the indirect CD133 MicroBead kit and LS columns (Miltenyi, Germany). Briefly, 80% confluent cells were washed with PBS and detached with Accutase (PAA, Austria) and adjusted up to 4×10^7^ cells per column and sorted with a modified PBS buffer containing 5% FBS (MACS buffer).

### Flow cytometry/Fluorescence assisted cell sorting (FACS)

Cells were harvested using Accutase, pelleted at 1000 rpm and re-suspended in MACS buffer. 2.5×10^5^ cells were incubated either with human specific CD271-PE or CD271-APC antibody (Miltenyi, clone ME20.4-1.H4, mouse IgG1, 1∶20), human specific CD133 antibody (Miltenyi, clone W6B3C1, mouse IgG1, 1∶10,) or mouse IgG1 Isotype control (Miltenyi) at 4°C for 10 min. Washed cells were re-suspended in 500 µl PBS and stored on ice or labeled with a secondary antibody. Cells were incubated in 100 µl buffer with AlexaFluor488, anti-mouse (Invitrogen).Stained cells were used for cell sorting or flow cytometry using FACSAria III or FACScalibur and Accuri C6 (Beckton&Dickinson), respectively. Data analysis was done with FlowJo software.

### Label-retaining assay

Labeling of cells using the fluorescent lipophilic dye PKH26 was done with accordance to the manufacturer's instructions. Labeled cells were re-plated and monitored. Analysis of co-labeling of PKH26 (PE), CD271 (APC) and CD133 (FITC) was done with Accuri C6. Analysis of labeled and sorted cells of each cell strain was performed in at least three independent experiments.

### Western-Blot

25 µg–40 µg of total protein lysates were separated on a 8% SDS-PAGE gel and transferred on a nitrocellulose membrane by using the iBlot Dry Blotting System (Invitrogen). Membranes were incubated with primary antibodies (CD271, clone D4B3, XP, Cell signaling; SOX10, clone 20B7, RnD; CD133, clone C24B9 and β-Tubulin, clone 9F3 both from Cell Signaling Technology, Germany; all diluted 1∶1000) overnight at 4°C and with a secondary antibody (goat anti-rabbit IgG LI-COR Biosciences, USA) for 1 h at RT. Detection was done with the Odyssey Infrared Imaging System (LI-COR Biosciences, USA).

### RNA isolation and RT-PCR

RNA isolation from frozen cell pellets was performed with the RNeasy Mini Kit (Qiagen, Germany) and from 5 µm FFPE sections using the RNeasy FFPE Kit (Qiagen), following the manufacturers protocol. Reverse transcription of 500 ng–2.5 µg RNA was performed with SuperScript VILO cDNA synthesis kit (Invitrogen, Germany) and diluted to a final volume of 50 µl. qRT-PCR was carried out on a Step one plus (Applied Biosystems, Germany) for 30–40 cycles. Primers were designed for 55–60°C annealing temperatures (Table S1). In qPCR expression levels were calculated either with ΔCT or ΔΔCT, normalized to β-actin. PCR products (15 µl) were separated on a 2% agarose (BioRad) gel.

### Tumorigenicity

Briefly, a number of 1×10^5^–1×10^6^ cells was mixed with Matrigel (1∶1) and subcutaneously injected in NSG [48] mice (n = 3/per group). Tumor volume given as [(tumor width)^2^ × tumor length] ×0.5 was measured with a caliper instrument and was monitored during the entire experiment with the measurements of two perpendicular tumor diameters.

### Ethics Statement about Animal Experiments

All animal experiments were performed at the EPO GmbH, Berlin, Germany in accordance with the German Animal Protection Law and approved by the local responsible authorities (LAGeSo A0452/08). The *in vivo* procedures were consistent and in compliance with the UKCCCR guidelines.

### Immunohistochemistry and Immunofluorescence analysis (IF)

FFPE material was sectioned to 2 µm after de-waxing, peroxidase blocking and antigen-retrieval using citrate buffer, pH 6.0. IHC for melanoma markers Melanosome (clone HMB45, mouse, 1∶50, Dako); Tyrosinase (clone T311, mouse, 1∶50, Novocastra); MITF (clones C5+D5, mouse, 1∶25, Zytomed) and CD271 (clone D4B3, XP, rabbit, 1∶100, Cell signaling), as well as hematoxylin/eosin (H&E) was done with the Discovery XT biomarker platform (Ventana). For IF on tumor sections: sections were blocked in 5% goat serum at RT for 2 h and incubated with primary antibodies: human specific CD133 (clone W6B3C1, mouse, 1∶10, Miltenyi); CD133 (polyclonal, rabbit 1∶100, Biorbyt); CD271 (clone D4B3-XP, rabbit, 1∶100, Cell signaling) and MART-1 (Melan-A clone A103/M2-7C10/M2-9E3, 1∶50, Zymed) diluted in 2% BSA/PBS. Incubation was done in a wet chamber overnight at 4°C. Sections washed with PBS/0.1% Tween-20 were incubated with secondary antibodies AlexaFluor488, anti-rabbit and AlexaFluor594, anti-mouse (both Invitrogen) for 1 h, at RT. IF on cultured cells: after washing with PBS and a fixation step (4% PFA in PBS for 10 min at RT), cells were either permeabilized with 0.1% Triton-X100 for staining of nuclear proteins or directly blocked with 2% BSA in PBS. Fixed cells were incubated with primary antibodies (CD271-PE and CD271-APC, Miltenyi, clone ME20.4-1.H4; SOX10, clone 20B7, RnD; CD133 Miltenyi, clone W6B3C1; γH2AX (phosphorylated (Ser139) H2AX, DNA-damage marker) and pSmad 2 clone 138D4, Cell signaling) overnight at 4°C. Next day, cells were washed and incubated with secondary antibodies AlexaFluor488 and AlexaFluor594, anti-rabbit/anti-mouse (both Invitrogen) and 4′,6-Diamidino-2-phenylindole dihydrochloride (DAPI), (Sigma-Aldrich) for 1 h, at RT, washed and analyzed.

### Knock-down and overexpression of CD271

For knock-down of CD271, melanoma cells were transfected with 2 µg of each shRNA plasmid (#2: 5′-ACAACCTCATCCCTGTCTATT-3′; #3: 5′-CCCGAGCACATAGA CTCCTTT-3′ and #4: 5′-CCGAGCACATAGACTCCTTTA-3′) or control shRNA (shCtl., 5′-GGAATCTCATTCGATGCATAC-3′; all from Qiagen) using Lipofectamine2000 (Invitrogen). Cells were selected with puromycin (10 µg/ml) over a period of two weeks. For over-expression melanoma cells were stably transfected with a plasmid expressing GFP-tagged human CD271 (RG207966, OriGene) and selected with G418 (300 µg/ml, PAA).

### Gene expression profiling

Whole genome expression profiling of CD271^k.d.^ and shCtl. cells was performed with three biological replicates. Illumina raw data of BeadChip HumanHT-12V4 platform were summarized via the BeadStudio without normalization and background correction. Follow-up processing was done via the R/Bioconductor environment. The lumi package was used for data annotation. Following statistical tests, data were quantile normalized.

### Accession numbers

The microarray analysis data have been deposited to the NCBI Gene Expression Omnibus (GEO) with accession number GSE52456.

### Statistical analysis

Significantly differentially expressed genes were identified by statistical tests using the bioconductor limma package [49]. The significance in terms of the false discovery rate (FDR), the q-value, was determined by the q-value package applied to several statistical tests (Student's t-test, Welch test and Wilcoxon test). Genes were defined significantly differentially expressed when the average Affymetrix (MAS5) detection p-value of at least one case (treatment or control) was <0.05 and one of the p- values of statistical tests (Student's t-test, Welch test and Wilcoxon test) was <0.05. Genes with ratios >1.33 or <0.75 were defined as significantly up-regulated or down-regulates, respectively. Heat maps were generated using Spearman's rank correlation as similarity measure and complete linkage. Genes with a limma false discovery rate (FDR) below (≤0.005) were defined as highly significantly regulated. Using this criteria we found 123 genes comprising 68 down-regulated (ratio <0.05) and 55 up-regulated genes (ratio >20). P-values depicting significance of experimental values were calculated by Excel using a two-tailed test.

### Meta-analysis

Whole genome expression profiling data of SOX10^k.d.^ cells stored at NCBI GEO, accession number GSE37059 were processed via the R/Bioconductor environment using statistical analysis tools mentioned above. Affymetrix chip data were processed via package affy and normalized with Robust Multi-array Average (RMA) from the same package. Package limma was used for making significance tests for differential gene expression. The results of these tests were adjusted for multiple testing via package q-value. Data were normalized with Robust Multi-array Average (RMA). Genes up-regulated (ratio >1.33) and down-regulated (ratio <0.75) with an average detection p-value <0.05 were analysed. Intersection sets were found via gene symbol annotations.

### Gene-Set enrichment Analysis (GSEA)

Quantile normalized expression data of CD271^k.d.^ cells and shCtl. cells were analysed with the GSEA analyses tool of the Broad institute (http://www.broadinstitute.org/gsea/index.jsp) and the molecular signature databases c2.all.v4.0.symbols.gmt (curated gene sets) and c3.all.v4.0.symbols.gmt (motif gene sets).

## Supporting Information

Figure S1 **The knock-down of CD271 induces strong changes in cellular morphology and gene-expression.** (**A**) Knock-down of CD271 in melanoma cells led to strong morphological changes indicated by rearrangement of the marker CD166 (ALCAM) as determined by immunofluorescence microscopy. Nuclei were stained with DAPI (blue), scale bars indicate 50 µm. Phase contrast (PH) depicts cellular morphology. (**B**) Deceleration of growth of melanoma cells stably transfected with shRNA plasmids #2, #3 or #4 (1×10^5^ cells plated). Cell counts ± SD of n = 3 experiments are shown. (**C**) Clustering of 7895 differentially regulated genes among biological triplicates of shCtl. vs. CD271^k.d.^ cells (shRNA#4) depicts 4048 (51.3%) down-regulated and 3874 (48.7%) up-regulated genes. By more stringent criteria these numbers changed to 68 down-regulated and 55 up-regulated genes. (**D**) Immunofluorescence microscopy of melanoma cells stably transfected with either shRNA plasmids #2, #3 and #4 or shRNA control (shCtl.) revealed efficient silencing of CD271 (upper panels) as well as a strong down-regulation of SOX10 (center panels) and FOXD3 (lower panels) by shRNA #3 and #4. Note the nuclear localization of SOX10 and FOXD3 in shCtl. and sh#2 cells and diffuse or absent staining in sh#3 and sh#4 stably transfected cells, respectively. Representative areas are shown. Nuclei were stained with DAPI (blue), scale bars indicate 50 µm. (**E**) Western blot of 25 µg of whole cell extracts of untransfected (Mock) or shRNA plasmid transfected cells for CD271 and SOX10. A representative out of three is shown. Tubulin served as loading control. (**F**) Melanoma cells that were stably transfected with a CD271 expression plasmid (CD271^exo^) showed an inverse correlation of CD271 and CD133. Shown are ΔΔCT values normalized to β-actin and related to expression levels in Mock cells as mean value ± SD of biological triplicates.
(TIF)

Figure S2 **CD271^k.d.^ cells show increased DNA-damage and decreased expression of anti-apoptotic genes.** (**A**) qPCR for expression levels of *CD271*, *CD133*, *SOX10*, *SOX2*, *ERBB3* and *FOXD3* in MeWo cells stably transfected with shRNA plasmids #2, #3 or #4. Expression levels of shRNA control (shCtl.) cells and of cells transfected with CD271-targeting shRNA plasmids are shown as ΔΔCT values normalized to β-actin and related to shCtl. cells as mean value ± SD of biological triplicates. The scale is logarithmic (log). (**B**) Immunofluorescence microscopy of MeWo cells transfected with either a shCtl. plasmid or shRNA#4 plasmid for CD271 and SOX10 reveal strong expression or efficient down-regulation of both proteins, respectively. Phase contrast (PH) depicts cellular morphology. (**C**) Immunofluorescence microscopy of MeWo cells transfected with shRNA plasmid #4 (sh#4) for SOX10 and CD166 showing their mutually exclusive expression. A representative out of three is shown. (**D**) Analysis of mRNA expression levels of anti-apoptotic genes cIAP1 (*BIRC2*), cIAP2 (*BIRC3*) and *BCL-2* in melanoma cells stably transfected with either shCD271 plasmids (sh#2, sh#3, sh#4) or shRNA control (shCtl.) by qPCR. mRNA expression levels are shown as ΔΔCT values normalized to β-actin and related to shCtl. cells as mean values ± SD of biological triplicates. *p≤0.05; **p≤0.01; ***p≤0.001 (t-test). (**E**) Detection of DNA-damage in melanoma cells transfected with either a shCtl. plasmid or shRNA plasmids #3 and #4 by immunofluorescence microscopy for γH2AX (arrows). In (C) and (E) Nuclei were stained with DAPI (blue), scale bars indicate 50 µm.
(TIF)

Figure S3 **CD271 but not CD133 is frequently expressed on melanoma cells.** (**A–D**) Flow cytometry of 10 patient-specific melanoma metastases-derived cell strains as well as (**E**) cell lines A375 and MeWo. Results show presence of distinct CD271^+^ and CD133^+^ populations in % or in % ± SD of biological triplicates. Mouse IgG1 served as isotype control. Tumor metastases represent three entities LN (n = 3); SKIN (n = 5); PUL (n = 2). Cell lines MeWo and A375 were established from a LN or SKIN metastasis, respectively. (**F**) Detection of CD133 protein level in CD133^+^ and CD133^−^ MACS-enriched cells by western blot analysis. Tubulin served as loading control.
(TIF)

Figure S4 **Slowly-dividing melanoma cells are dye-retaining.** (**A**) Immunofluorescence microscopy of melanoma cells (Mel9-1 and Mel4-7) for expression of CD271 or CD133, respectively. Nuclei were stained with DAPI (blue), scale bars indicate 50 µm. (**B–C**) PKH26 in dye-retaining cells 7 days and 14 days after labeling. Phase contrast (PH) depicts cellular morphology. Scale bars indicate 50 µm. (**D**) Corresponding isotype controls depict negative cells for analysis of dye-retaining cells for presence of CD271 and CD133.
(TIF)

Figure S5 **CD271^+^ cells re-establish cellular heterogeneity** ***in vitro*** **.** (**A**) FACS-plot indicates the distribution of CD271^+^, CD133^+^ and CD271^+^/CD133^+^ cells before FACS, the area of CD271^+^ cells is highlighted (red border, left panel).Yield of CD271^+^ FACS-sorted cells determined by flow cytometry after sorting (center panel) in comparison to mouse IgG1 isotype control (right panel). (**B**) Immunofluorescence microscopy of CD271^+^ cells, 3 days after sorting for expression of CD133 and CD271. (**C**) Amount of cells analyzed in (B). Bars indicate mean values ± SD of n = 8 counts. (**D**) Isolation of CD271/CD133 double positive cells (red rectangle) by FACS (center panel). Re-analysis of sorted and cultured cells after 7 days shows the derivation of CD133^+^/CD271^−^ cells from CD271/CD133 double positive cells. (**E**) Summary of flow cytometry results shows presence of cells in distinct CD271^+^ and CD133^+^ or CD271/CD133 double positive or double negative sub-fractions indicating the time-dependent increase of CD133^+^ cells. Sub-sets are represented as % ± SD of biological triplicates. Mouse IgG1 served as isotype control. ***p≤0.001 (t-test). (**F**) Analysis of PKH26-labeled double positive cells following 3 days and 7 days of isolation by immunofluorescence microscopy reveals the presence of dye-retaining cells.
(TIF)

Figure S6 **TGFβ represses SOX10 expression in melanoma cells.** (**A**) Validation of cellular plasticity with a different melanoma cell strain. Cells were sorted either for a CD271^+^/CD133^−^ phenotype or absence of both markers. (**B**) Flow cytometry of cells following isolation and cultivation after 3 days for cellular sub-sets proves the re-establishment of the cellular heterogeneity of the initial cell culture irrespective of their phenotype. (**C**) Phase contrast images of unsorted and sorted cells depict their specific cellular morphology. (**D**) Verification of flow cytometry results by immunofluorescence microscopy. Single and double positive cells were observed in the cell culture 3 d after sorting. (**E**) Immunofluoresecence microscopy of shCtl. cells untreated (Ctl.) or treated with TGFβ2 (10 ng/ml) for 3 days for levels of phospho-Smad 2 (pSmad2) depicting active TGFβ-signaling, SOX10 and CD133. TGFβ2-treatment led to a strong decrease of SOX10 whereas CD133 was not affected. Nuclei were stained with DAPI (blue), scale bars indicate 50 µm.
(TIF)

Figure S7 **CD271 and differentiation markers are mutually exclusive expressed in melanoma metastases.** (**A**) Summary of patient cohort, tumor site, first metastasis and final metastasis. ALM: acral lentiginous melanoma; SSM: superficial spreading melanoma; UVE: uveal melanoma; MUC: mucosal melanoma. LN: lymph node; HEP: hepatic; PUL: lung; SKIN: cutaneous metastases. The table also sums up the expression of markers from IHC. (**B**) Mutually exclusive expression of CD271 and differentiation markers TYR, HMB45 and MITF in cutaneous and (**C**) hepatic metastases as indicated.
(TIF)

Figure S8 **Comparison of melanoma metastases and xenograft tumors.** (**A**) Comparison of a primary metastasis (PM) of patients T20/19 and (**B**) T20/20 and the corresponding xenografted tumor (PMX) for presence and localization of CD271 and MITF shows the mutually exclusive expression of these proteins in both tumor entities, marked by the red dashed line. (**C**) Heterogeneity was also established by cultured cells of patient T20/02 upon tumor formation. Expression patterns of CD271 and MITF are comparable to those observed in the primary metastasis. Scale bars indicate 50 µm.
(TIF)

Figure S9 **TGFβ1 induced genes are enriched in CD271^k.d.^ cells.** (**A–B**) Comparison of data sets of shCtl. and CD271^k.d.^ cells with data sets of murine mammary epithelium cells (NMuMG cells) after stimulation with TGFβ1 [53] by gene-set enrichment analysis (GSEA). GSEA revealed the enrichment of TGFβ1 induced genes like *CTGF*, *SMAD7*, *TGIF* and *SERPINE1* upon CD271 silencing.
(TIF)

Table S1 **qPCR primers.** Primers were designed with primer quest at http://eu.idtdna.com/site with an average of 22 bp and an annealing temperature of 60°C yielding in products of 150–250 bp. Details about qPCR can be found in the materials and methods section.
(DOCX)

Table S2 **Statistics of grown Tumors.** The volume of tumors, grown subcutaneously in NSG mice was determined once a week.
(DOCX)
