# Correction: The Nerve Growth Factor Receptor CD271 Is Crucial to Maintain Tumorigenicity and Stem-Like Properties of Melanoma Cells

**DOI:** 10.1371/journal.pone.0105274

**Published:** 2014-08-04

**Authors:** The PLOS ONE Staff

There are errors in some of the authors' affiliations listed for this article. Please refer to the correct affiliations below:

Torben Redmer, Yvonne Welte, Dorothea Przybilla, Wasco Wruck, Reinhold Schäfer, Christian R. A. Regenbrecht

1 Institute for Pathology, Charité - Universitätsmedizin Berlin, Berlin, Germany

Diana Behrens, Iduna Fichtner

2 Experimental Pharmacology & Oncology Berlin-Buch GmbH, Berlin, Germany

Wasco Wruck, Christian R. A. Regenbrecht

3 Laboratory of Functional Genomics (LFGC), Charité - Universitätsmedizin Berlin, Berlin, Germany

Marie-Laure Yaspo, Hans Lehrach

4 Max-Planck Institute for Molecular Genetics, Berlin, Germany

Dorothea Przybilla, Reinhold Schäfer, Christian R. A. Regenbrecht

5 Comprehensive Cancer Center Charité, Charité - Universitätsmedizin Berlin, Berlin, Germany
