# Supplementary figures and images for: The Nerve Growth Factor Receptor CD271 Is Crucial to Maintain Tumorigenicity and Stem-Like Properties of Melanoma Cells
Source: PLoS One. 2014 May 5;9(5):e92596. doi: 10.1371/journal.pone.0092596 (PMC4010406; doi:10.1371/journal.pone.0092596)

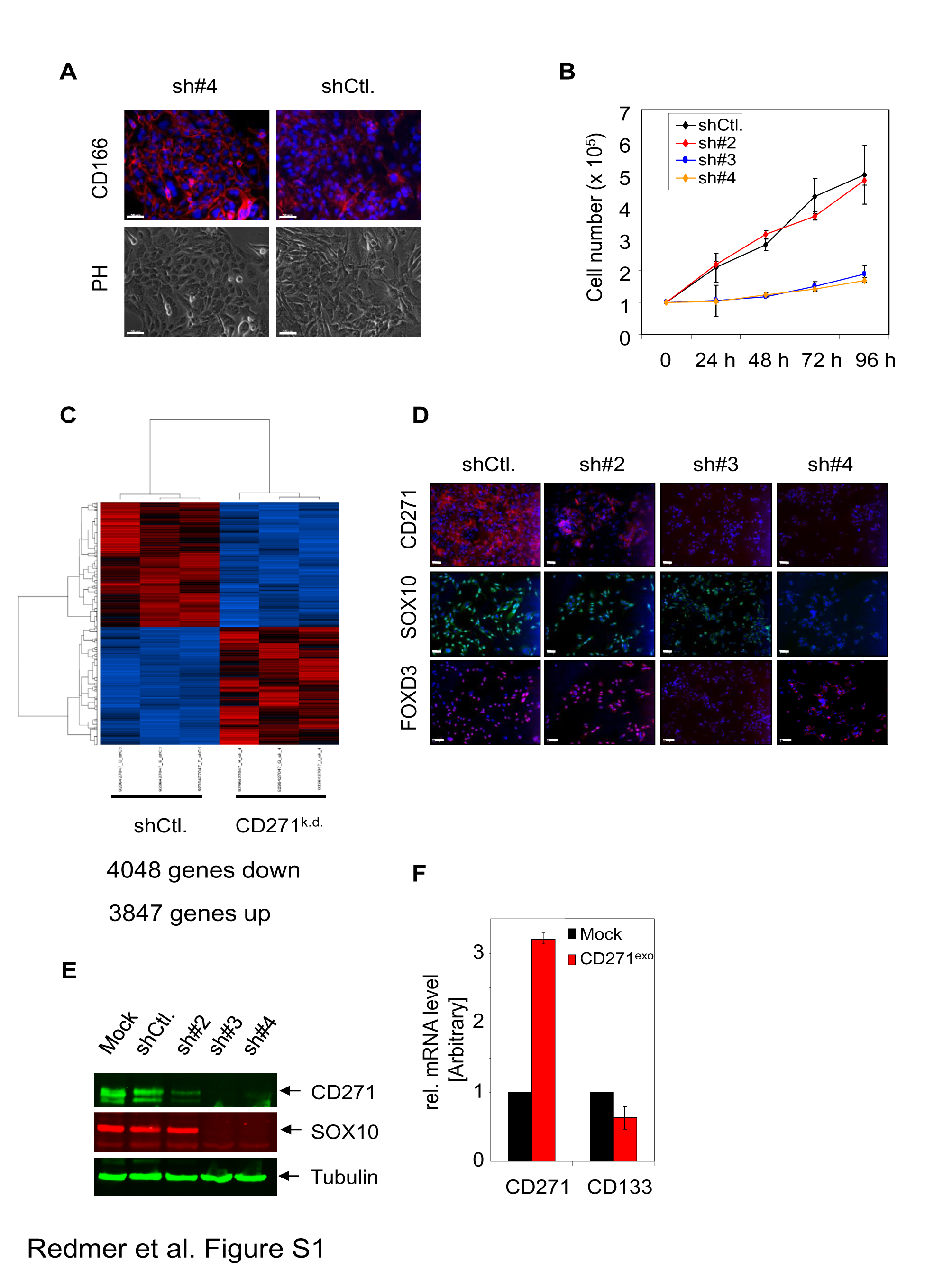

Supplement: Figure S1 — The knock-down of CD271 induces strong changes in cellular morphology and gene-expression. (A) Knock-down of CD271 in melanoma cells led to strong morphological changes indicated by rearrangement of the marker CD166 (ALCAM) as determined by immunofluorescence microscopy. Nuclei were stained with DAPI (blue), scale bars indicate 50 µm. Phase contrast (PH) depicts cellular morphology. (B) Deceleration of growth of melanoma cells stably transfected with shRNA plasmids #2, #3 or #4 (1×10^5^ cells plated). Cell counts ± SD of n = 3 experiments are shown. (C) Clustering of 7895 differentially regulated genes among biological triplicates of shCtl. vs. CD271k.d. cells (shRNA#4) depicts 4048 (51.3%) down-regulated and 3874 (48.7%) up-regulated genes. By more stringent criteria these numbers changed to 68 down-regulated and 55 up-regulated genes. (D) Immunofluorescence microscopy of melanoma cells stably transfected with either shRNA plasmids #2, #3 and #4 or shRNA control (shCtl.) revealed efficient silencing of CD271 (upper panels) as well as a strong down-regulation of SOX10 (center panels) and FOXD3 (lower panels) by shRNA #3 and #4. Note the nuclear localization of SOX10 and FOXD3 in shCtl. and sh#2 cells and diffuse or absent staining in sh#3 and sh#4 stably transfected cells, respectively. Representative areas are shown. Nuclei were stained with DAPI (blue), scale bars indicate 50 µm. (E) Western blot of 25 µg of whole cell extracts of untransfected (Mock) or shRNA plasmid transfected cells for CD271 and SOX10. A representative out of three is shown. Tubulin served as loading control. (F) Melanoma cells that were stably transfected with a CD271 expression plasmid (CD271exo) showed an inverse correlation of CD271 and CD133. Shown are ΔΔCT values normalized to β-actin and related to expression levels in Mock cells as mean value ± SD of biological triplicates. (TIF) [file pone.0092596.s001.tif]

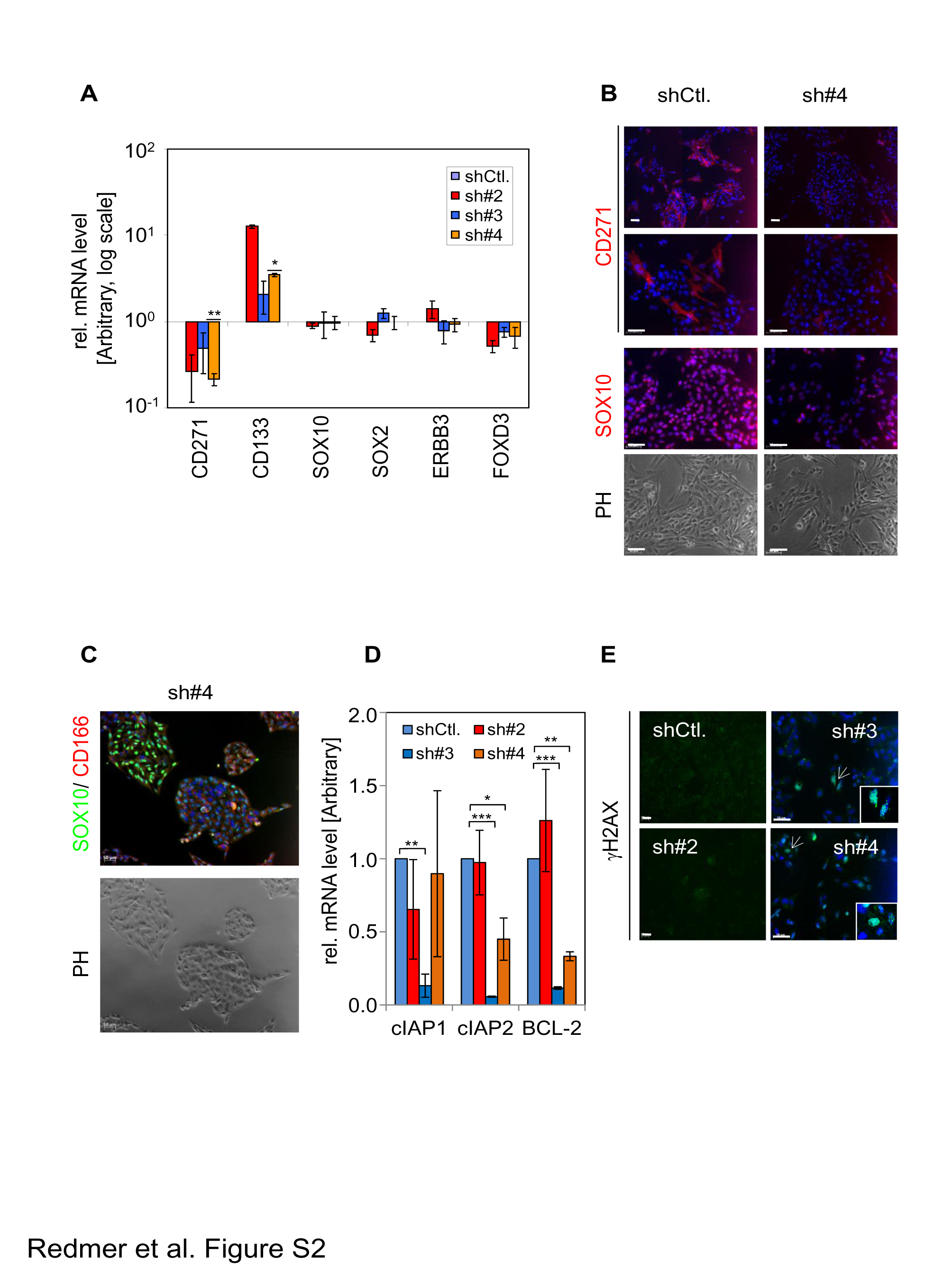

Supplement: Figure S2 — CD271k.d. cells show increased DNA-damage and decreased expression of anti-apoptotic genes. (A) qPCR for expression levels of CD271, CD133, SOX10, SOX2, ERBB3 and FOXD3 in MeWo cells stably transfected with shRNA plasmids #2, #3 or #4. Expression levels of shRNA control (shCtl.) cells and of cells transfected with CD271-targeting shRNA plasmids are shown as ΔΔCT values normalized to β-actin and related to shCtl. cells as mean value ± SD of biological triplicates. The scale is logarithmic (log). (B) Immunofluorescence microscopy of MeWo cells transfected with either a shCtl. plasmid or shRNA#4 plasmid for CD271 and SOX10 reveal strong expression or efficient down-regulation of both proteins, respectively. Phase contrast (PH) depicts cellular morphology. (C) Immunofluorescence microscopy of MeWo cells transfected with shRNA plasmid #4 (sh#4) for SOX10 and CD166 showing their mutually exclusive expression. A representative out of three is shown. (D) Analysis of mRNA expression levels of anti-apoptotic genes cIAP1 (BIRC2), cIAP2 (BIRC3) and BCL-2 in melanoma cells stably transfected with either shCD271 plasmids (sh#2, sh#3, sh#4) or shRNA control (shCtl.) by qPCR. mRNA expression levels are shown as ΔΔCT values normalized to β-actin and related to shCtl. cells as mean values ± SD of biological triplicates. *p≤0.05; **p≤0.01; ***p≤0.001 (t-test). (E) Detection of DNA-damage in melanoma cells transfected with either a shCtl. plasmid or shRNA plasmids #3 and #4 by immunofluorescence microscopy for γH2AX (arrows). In (C) and (E) Nuclei were stained with DAPI (blue), scale bars indicate 50 µm. (TIF) [file pone.0092596.s002.tif]

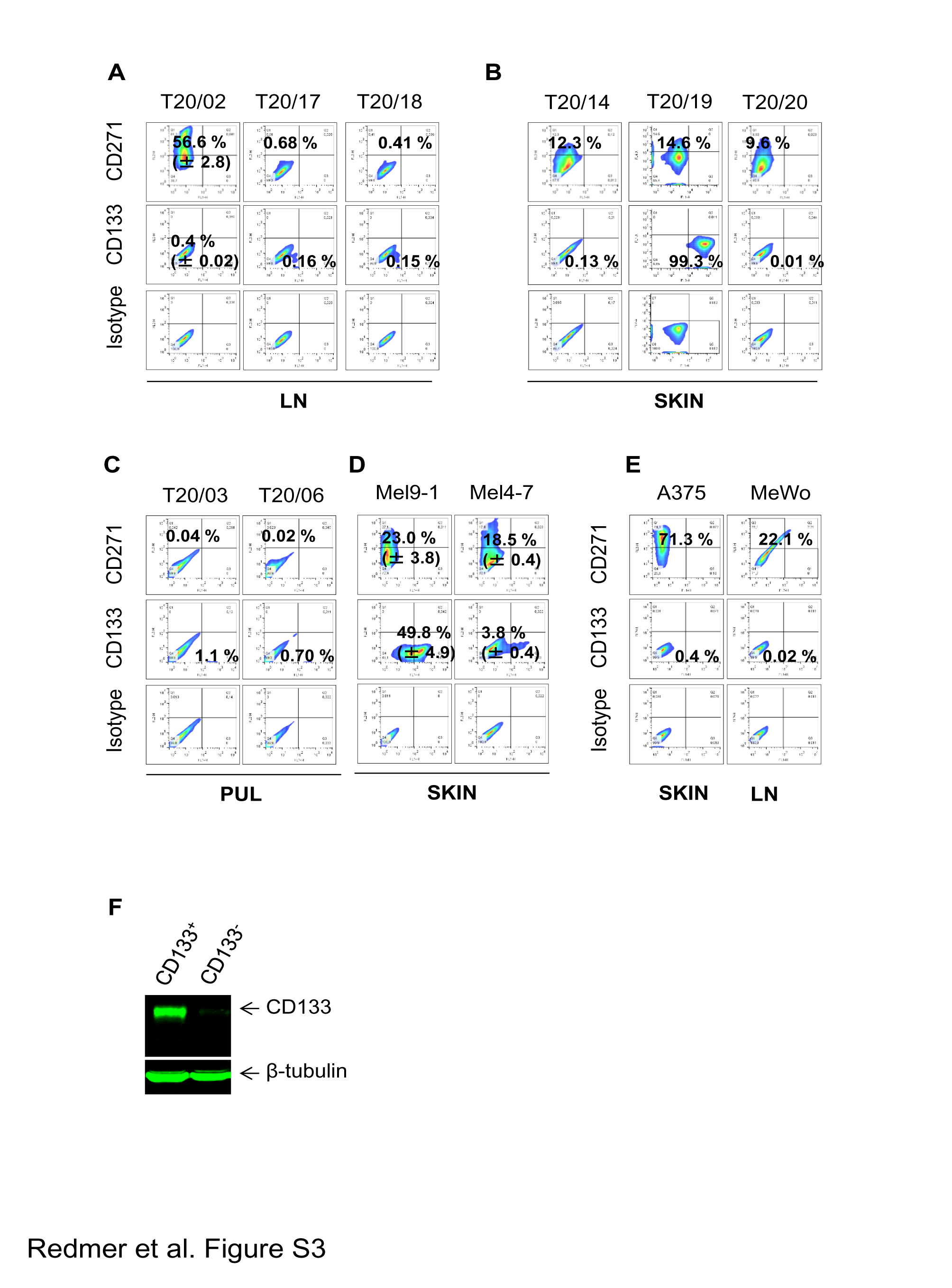

Supplement: Figure S3 — CD271 but not CD133 is frequently expressed on melanoma cells. (A–D) Flow cytometry of 10 patient-specific melanoma metastases-derived cell strains as well as (E) cell lines A375 and MeWo. Results show presence of distinct CD271+ and CD133+ populations in % or in % ± SD of biological triplicates. Mouse IgG1 served as isotype control. Tumor metastases represent three entities LN (n = 3); SKIN (n = 5); PUL (n = 2). Cell lines MeWo and A375 were established from a LN or SKIN metastasis, respectively. (F) Detection of CD133 protein level in CD133+ and CD133− MACS-enriched cells by western blot analysis. Tubulin served as loading control. (TIF) [file pone.0092596.s003.tif]

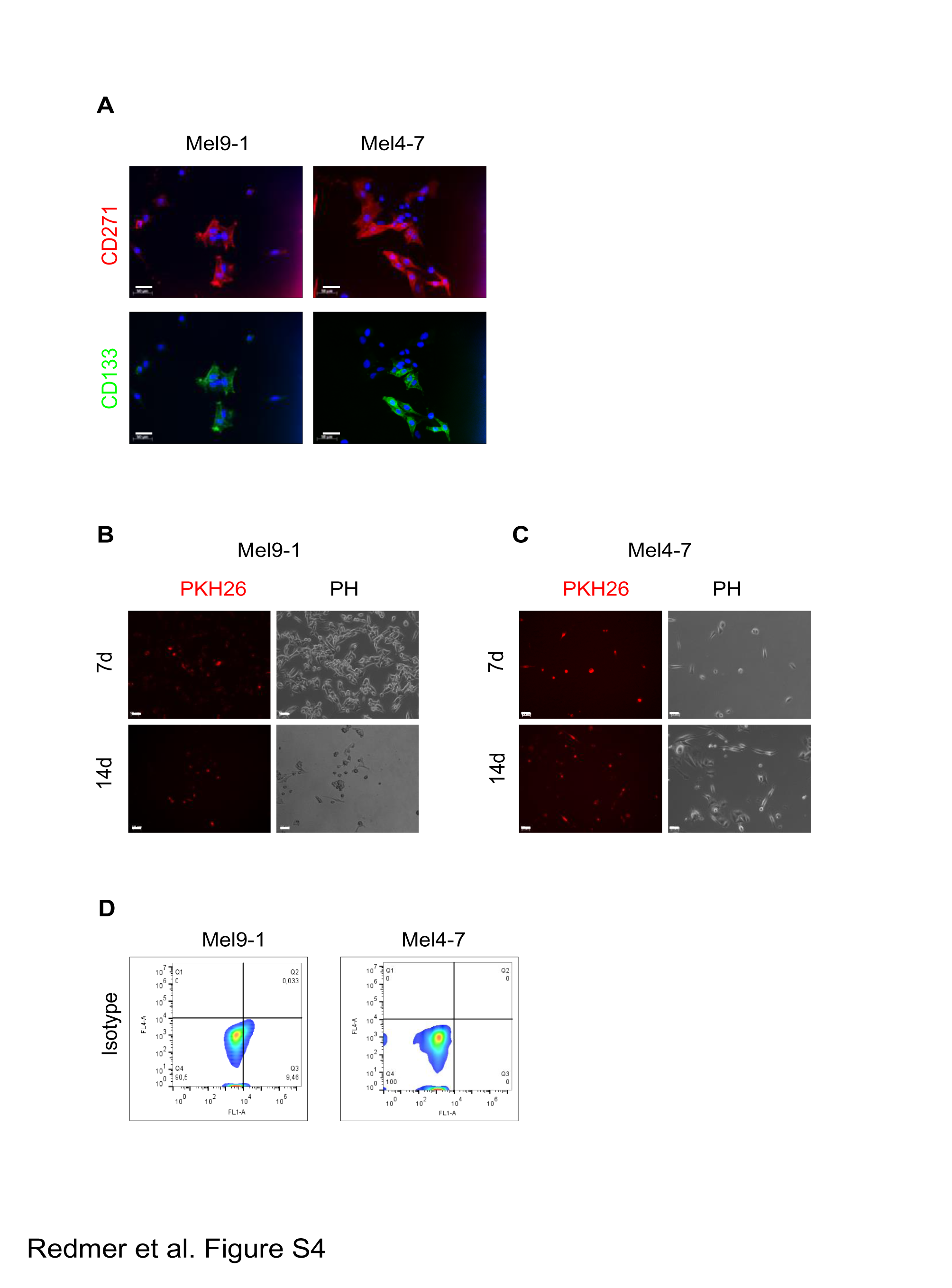

Supplement: Figure S4 — Slowly-dividing melanoma cells are dye-retaining. (A) Immunofluorescence microscopy of melanoma cells (Mel9-1 and Mel4-7) for expression of CD271 or CD133, respectively. Nuclei were stained with DAPI (blue), scale bars indicate 50 µm. (B–C) PKH26 in dye-retaining cells 7 days and 14 days after labeling. Phase contrast (PH) depicts cellular morphology. Scale bars indicate 50 µm. (D) Corresponding isotype controls depict negative cells for analysis of dye-retaining cells for presence of CD271 and CD133. (TIF) [file pone.0092596.s004.tif]

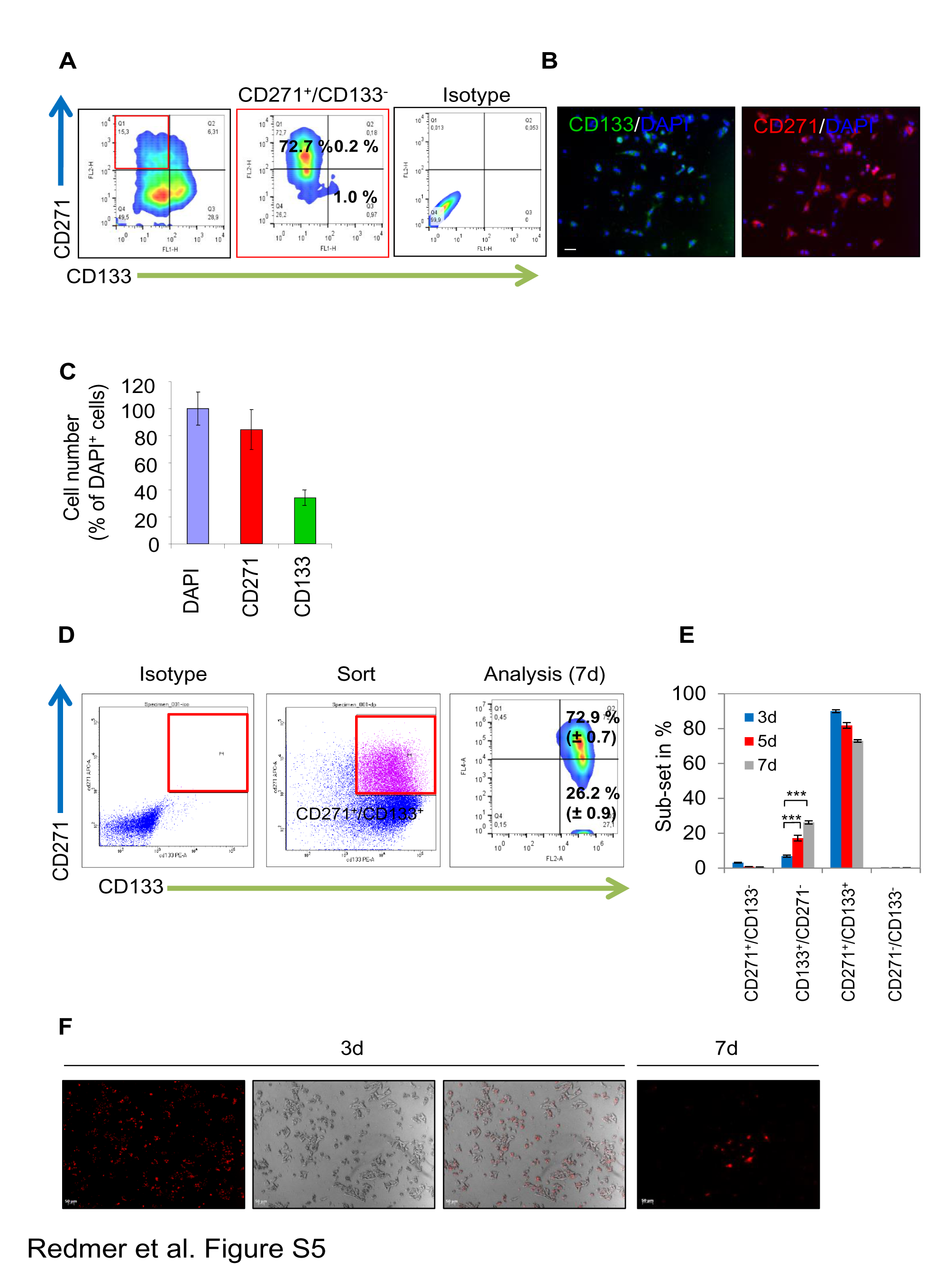

Supplement: Figure S5 — CD271+ cells re-establish cellular heterogeneity in vitro . (A) FACS-plot indicates the distribution of CD271+, CD133+ and CD271+/CD133+ cells before FACS, the area of CD271+ cells is highlighted (red border, left panel).Yield of CD271+ FACS-sorted cells determined by flow cytometry after sorting (center panel) in comparison to mouse IgG1 isotype control (right panel). (B) Immunofluorescence microscopy of CD271+ cells, 3 days after sorting for expression of CD133 and CD271. (C) Amount of cells analyzed in (B). Bars indicate mean values ± SD of n = 8 counts. (D) Isolation of CD271/CD133 double positive cells (red rectangle) by FACS (center panel). Re-analysis of sorted and cultured cells after 7 days shows the derivation of CD133+/CD271− cells from CD271/CD133 double positive cells. (E) Summary of flow cytometry results shows presence of cells in distinct CD271+ and CD133+ or CD271/CD133 double positive or double negative sub-fractions indicating the time-dependent increase of CD133+ cells. Sub-sets are represented as % ± SD of biological triplicates. Mouse IgG1 served as isotype control. ***p≤0.001 (t-test). (F) Analysis of PKH26-labeled double positive cells following 3 days and 7 days of isolation by immunofluorescence microscopy reveals the presence of dye-retaining cells. (TIF) [file pone.0092596.s005.tif]

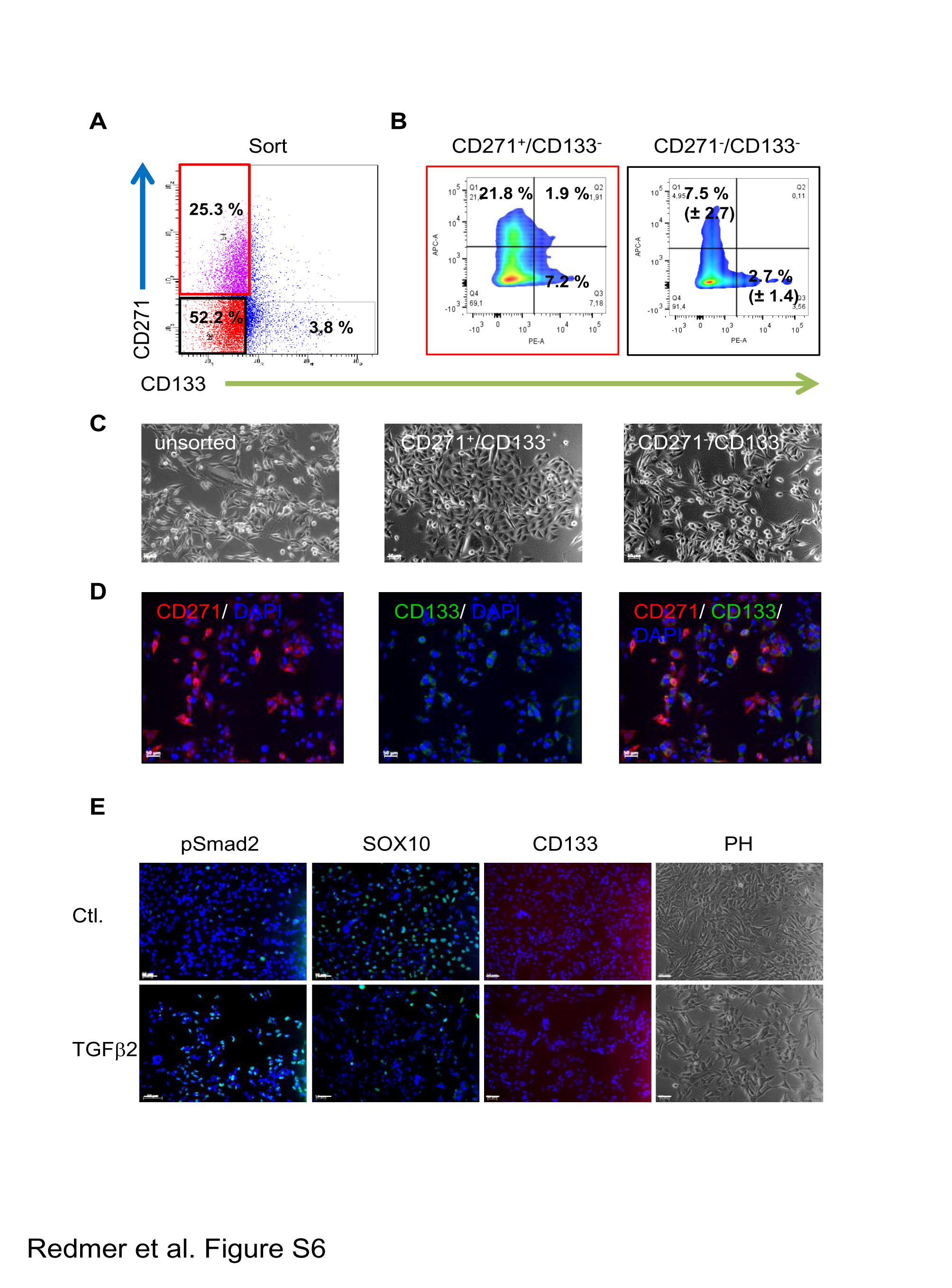

Supplement: Figure S6 — TGFβ represses SOX10 expression in melanoma cells. (A) Validation of cellular plasticity with a different melanoma cell strain. Cells were sorted either for a CD271+/CD133− phenotype or absence of both markers. (B) Flow cytometry of cells following isolation and cultivation after 3 days for cellular sub-sets proves the re-establishment of the cellular heterogeneity of the initial cell culture irrespective of their phenotype. (C) Phase contrast images of unsorted and sorted cells depict their specific cellular morphology. (D) Verification of flow cytometry results by immunofluorescence microscopy. Single and double positive cells were observed in the cell culture 3 d after sorting. (E) Immunofluoresecence microscopy of shCtl. cells untreated (Ctl.) or treated with TGFβ2 (10 ng/ml) for 3 days for levels of phospho-Smad 2 (pSmad2) depicting active TGFβ-signaling, SOX10 and CD133. TGFβ2-treatment led to a strong decrease of SOX10 whereas CD133 was not affected. Nuclei were stained with DAPI (blue), scale bars indicate 50 µm. (TIF) [file pone.0092596.s006.tif]

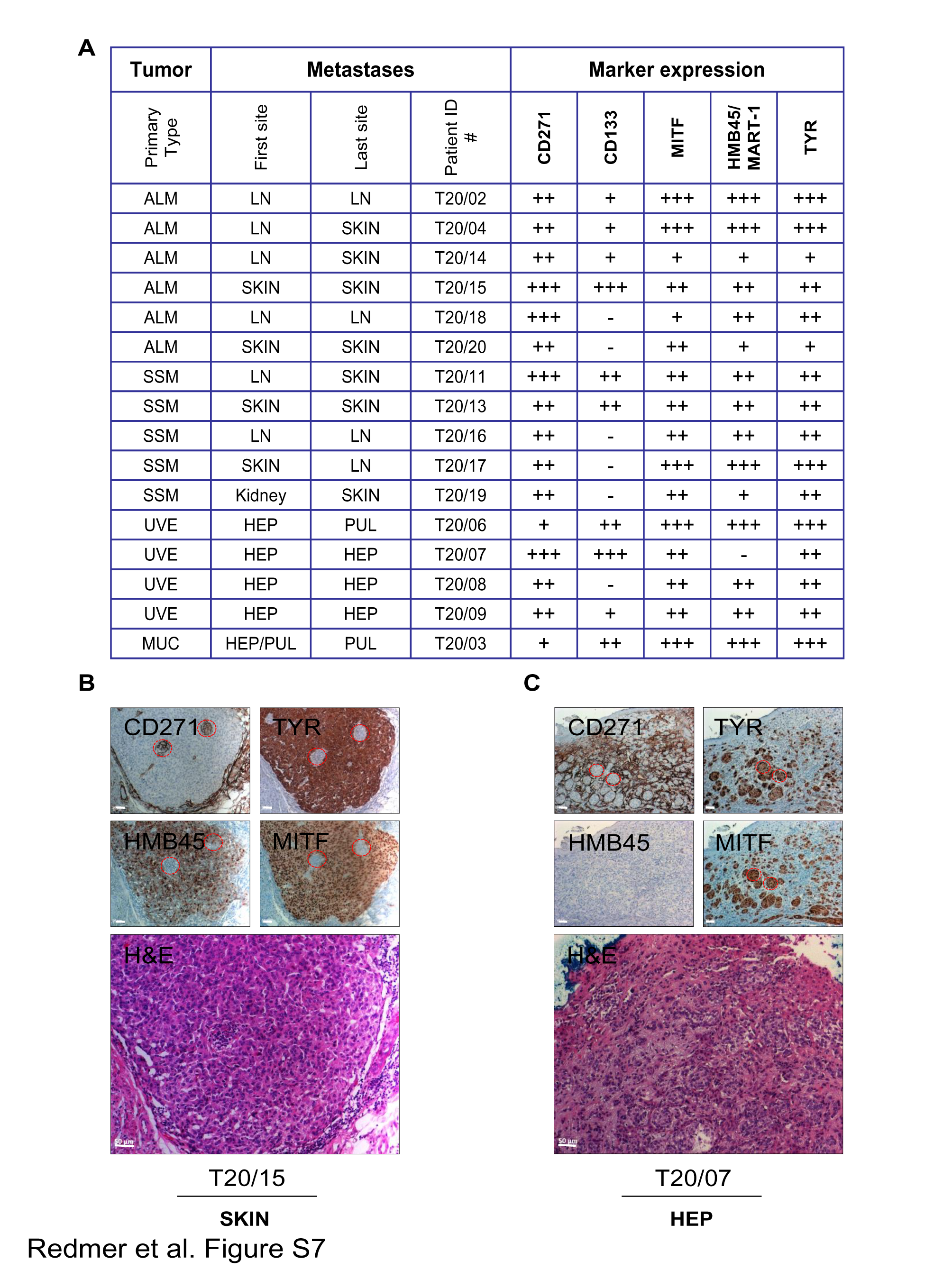

Supplement: Figure S7 — CD271 and differentiation markers are mutually exclusive expressed in melanoma metastases. (A) Summary of patient cohort, tumor site, first metastasis and final metastasis. ALM: acral lentiginous melanoma; SSM: superficial spreading melanoma; UVE: uveal melanoma; MUC: mucosal melanoma. LN: lymph node; HEP: hepatic; PUL: lung; SKIN: cutaneous metastases. The table also sums up the expression of markers from IHC. (B) Mutually exclusive expression of CD271 and differentiation markers TYR, HMB45 and MITF in cutaneous and (C) hepatic metastases as indicated. (TIF) [file pone.0092596.s007.tif]

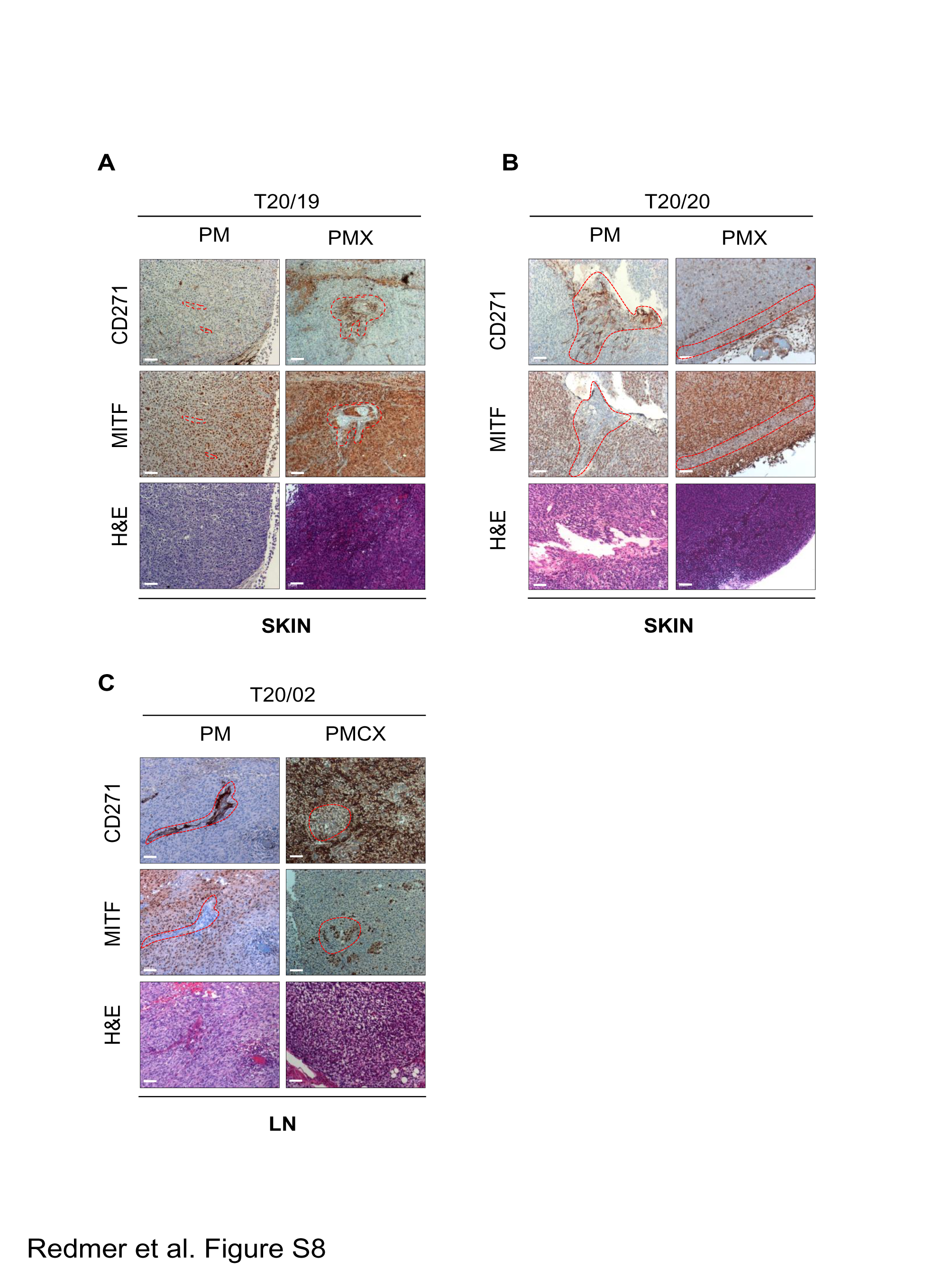

Supplement: Figure S8 — Comparison of melanoma metastases and xenograft tumors. (A) Comparison of a primary metastasis (PM) of patients T20/19 and (B) T20/20 and the corresponding xenografted tumor (PMX) for presence and localization of CD271 and MITF shows the mutually exclusive expression of these proteins in both tumor entities, marked by the red dashed line. (C) Heterogeneity was also established by cultured cells of patient T20/02 upon tumor formation. Expression patterns of CD271 and MITF are comparable to those observed in the primary metastasis. Scale bars indicate 50 µm. (TIF) [file pone.0092596.s008.tif]

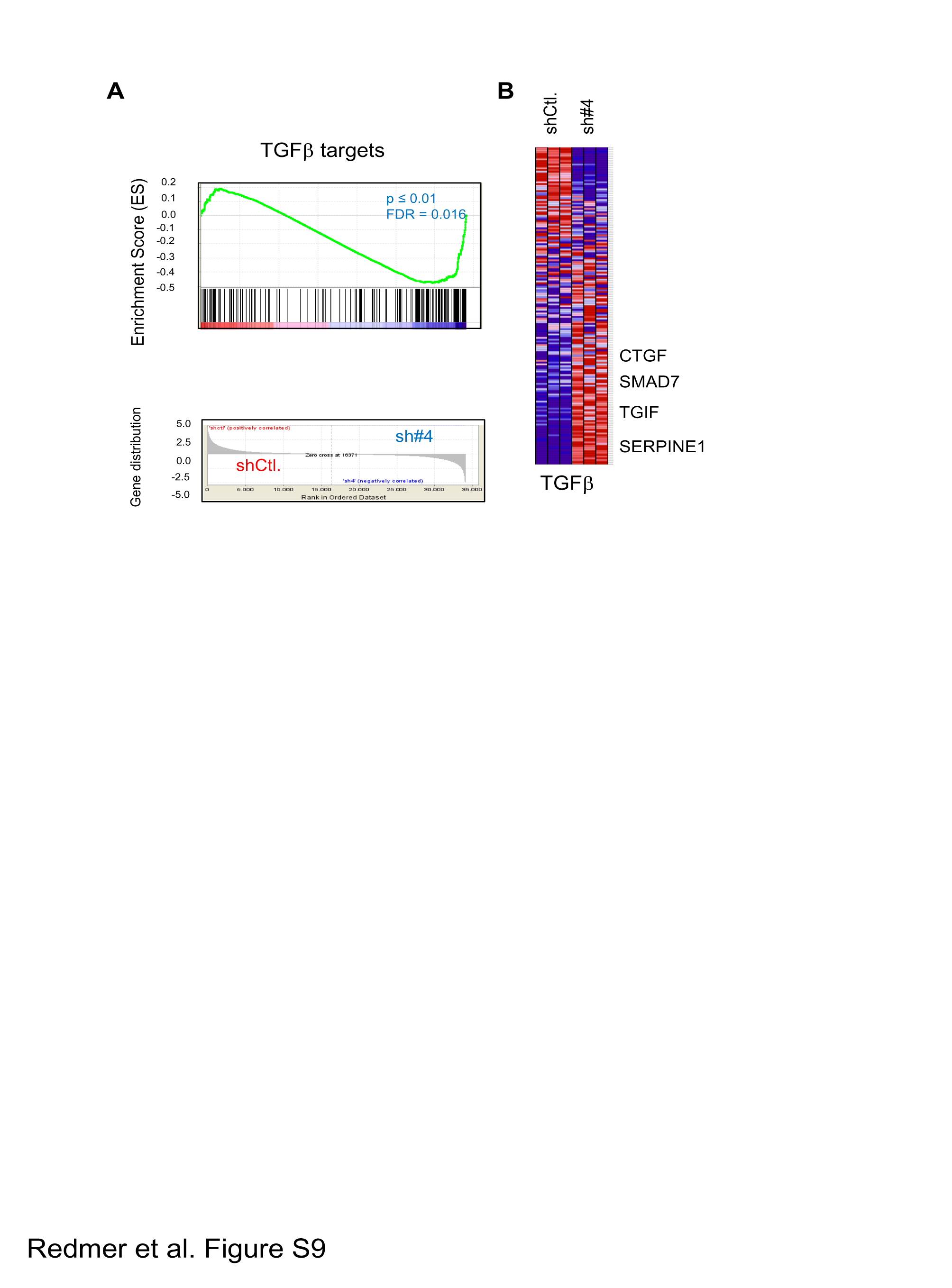

Supplement: Figure S9 — TGFβ1 induced genes are enriched in CD271k.d. cells. (A–B) Comparison of data sets of shCtl. and CD271k.d. cells with data sets of murine mammary epithelium cells (NMuMG cells) after stimulation with TGFβ1 [53] by gene-set enrichment analysis (GSEA). GSEA revealed the enrichment of TGFβ1 induced genes like CTGF, SMAD7, TGIF and SERPINE1 upon CD271 silencing. (TIF) [file pone.0092596.s009.tif]
