# Supplementary material for: The Nerve Growth Factor Receptor CD271 Is Crucial to Maintain Tumorigenicity and Stem-Like Properties of Melanoma Cells
Source: PLoS One. 2014 May 5;9(5):e92596. doi: 10.1371/journal.pone.0092596 (PMC4010406; doi:10.1371/journal.pone.0092596)
Supplement: Table S1 — qPCR primers. Primers were designed with primer quest at http://eu.idtdna.com/site with an average of 22 bp and an annealing temperature of 60°C yielding in products of 150–250 bp. Details about qPCR can be found in the materials and methods section. (DOCX) [file pone.0092596.s010.docx]

| Gene | Sequence | Accession Numbers |
| --- | --- | --- |
| ABCB5-s  ABCB5-as | CAATGGCCTTTTGTGGTTCTG  CCCTGCTCTGCCGTAAAATA | [GenBank:NM_001163941.1] |
| β-Actin-s  β-Actin-as | AGGAGAAGCTGTGCTACGTC  CTCGTCATACTCCTGCTTGC | [GenBank:NM_001199954.1] |
| BCL-2s-  BCL-2-as | GATTGTGGCCTTCTTTGAGTTCGG  TACAGTTCCACAAAGGCATCCCAGC | [GenBank:NM_000633 |
| CD271-s  CD271-as | ACCTCCAGAACAAGACCTCATAGC  TTGTTCTGCTTGCAGCTGTTCC | [GenBank:NM_002507] |
| CD133-s  CD133-as | CCTGGGGCTGCTGTTTATTA  TTGATCCGGGTTCTTACCTG | [GenBank:NM_006017] |
| cIAP1-s  cIAP1-as | AATGCTTCTGTTGTGGCCTGATGC  TCTGGTTTCAGCTAGTCTGGGATCCA | [GenBank:NM_001166] |
| cIAP-2-s  cIAP-2-as | TCCGTCAAGTTCAAGCCAGTTACC  TTCCACGGCAGCATTAATCACAGG | [GenBank:NM_001165] |
| ERBB3-s  ERBB3-as | GTGATAGCAGGATTGGTAGTG  GTTCCAAGTATCGCCTCATAG | [GenBank:NM_001982] |
| ETS1-s  ETS1-as | TCAAACAAGAAGTCGTCACC  ATAGTCCTCTGAGTCGAAGC | [GenBank:NM_001143820] |
| GLI-2-s  GLI-2-as | TGGACAGGGATGACTGTAA  CATGTACTGCGCCTTGAA | [GenBank:NM_005270] |
| HPRT-s  HPRT-as | GTTGTAGGATATGCCCTTGAC  GCCCAAAGGGAACTGATAGT | [GenBank:NM_000194.2] |
| IGFBP-2-s  IGFBP-2-as | TGGACAGGGATGACTGTAA  CATGTACTGCGCCTTGAA | [GenBank:NM_000597] |
| MART-1-s  MART-1-as | ACAGTGATCCTGGGAGTCTTACTGC  TTTGCTGTCCCGATGATCAAACCC | [GenBank:NM_005511.1] |
| MITF-M-s  MITF-M-as | TTATAGTACCTTCTCTTTGCCAGTCC  GTTTATTTGCTAAAGTGGTAGAAAGGTACT | [GenBank:NM_000248.3] |
| MYB-s  MYB-as | CTCCAGTCATGTTCCATACC  TGTGTGGTTCTGTGTTGG | [GenBank:NM_005375] |
| NES-s  NES-as | TGCTTACCACTTTGCCCTCT  TCCAAGACTTCCCTCAGCTT | [GenBank:NM_006617.1] |
| NFκB1-s  NFκB1-as | GGTGGGATTACTTTCCATCC  TAGAGTGACCTCACCATTCC | [GenBank:NM_003998] |
| RHOJ-s  RHOJ-as | GATCTGCTTCTCTGTCGTAAA  TTCACACCATGCTCGTAAG | [GenBank:NM_020663] |
| SOX2-s  SOX2-as | CAGCTCGCAGACCTACATGA  TGGAGTGGGAGGAAGAGGTA | [GenBank:NM_003106] |
| SOX5-s  SOX5-as | GCAACACCAGGCTTAGG  GTCTTGGGTTTAGCTGATAGG | [GenBank:NM_152989] |
| SOX6-s  SOX6-as | GAGGCAGTTCTTTACTGTGG  CGCCATCTGTCTTCATACC | [GenBank:NM_017508] |
| SOX8-s  SOX8-as | AACGCATTCATGGTGTGG  TGGCTGGTACTTGTAGTCG | [GenBank:NM_014587] |
| SOX10-s  SOX10-as | TTTGACTACTCTGACCATCAGCCC  AGTGTCGTATATACTGGCTGCTCC | [GenBank:NM_006941] |
| TFAP2A-s  TFAP2A-as | CGAAACCGAATTTCCTGCCAAAGC  AGATGAGGTTGAAGTGGGTCAAGC | [GenBank:NM_003220] |
| TFAP2C-s  TFAP2C-as | AGAAGTTGGACAAGATTGGG  AGCTGCCATCTCATTTCG | [GenBank:NM_003222] |
| TYR-s  TYR-as | ACCGGGAATCCTACATGGTT  TGAGGAGTGGCTGCTTTTCT | [GenBank:NM_000372] |
