# Supplementary material for: The Nerve Growth Factor Receptor CD271 Is Crucial to Maintain Tumorigenicity and Stem-Like Properties of Melanoma Cells
Source: PLoS One. 2014 May 5;9(5):e92596. doi: 10.1371/journal.pone.0092596 (PMC4010406; doi:10.1371/journal.pone.0092596)
Supplement: Table S2 — Statistics of grown Tumors. The volume of tumors, grown subcutaneously in NSG mice was determined once a week. (DOCX) [file pone.0092596.s011.docx]

| **Time [d]** | **10** | **15** | **22** | **29** | **36** | **43** | **50** | **52** | **57** | **Cell fraction** |
| --- | --- | --- | --- | --- | --- | --- | --- | --- | --- | --- |
| **Volume Tumor 1** | 0.001 | 0.001 | 0.004 | 0.092 | 0.181 | 0.379 | 0.707 | 0.825 | 1.269 | **CD271^+^** |
| **Volume Tumor 2** | 0.001 | 0.001 | 0.066 | 0.093 | 0.268 | 0.483 | 0.802 | 1.068 | 1.745 |  |
| **Volume Tumor 3** | 0.001 | 0.001 | 0.001 | 0.001 | 0.001 | 0.001 | 0.100 | 0.254 | 0.294 |  |
| **Median volume** | 0.001 | 0.001 | 0.024 | 0.062 | 0.150 | 0.288 | 0.536 | 0.716 | 1.103 |  |
| **sdv** | 0.000 | 0.000 | 0.037 | 0.053 | 0.136 | 0.254 | 0.381 | 0.418 | 0.740 |  |
| **Time [d]** | **8** | **15** | **21** | **28** | **34** | **42** | **49** | **55** | **62** | **Cell fraction** |
| **Volume Tumor 1** | 0.001 | 0.004 | 0.004 | 0.033 | 0.073 | 0.107 | 0.245 | 0.500 | 0.752 | **unsorted** |
| **Volume Tumor 2** | 0.001 | 0.004 | 0.004 | 0.027 | 0.064 | 0.111 | 0.123 | 0.469 | 0.718 |  |
| **Volume Tumor 3** | 0.001 | 0.004 | 0.004 | 0.036 | 0.049 | 0.075 | 0.182 | 0.392 | 0.758 |  |
| **Median volume** | 0.001 | 0.004 | 0.004 | 0.032 | 0.062 | 0.098 | 0.183 | 0.454 | 0.743 |  |
| **sdv** | 0.000 | 0.000 | 0.000 | 0.005 | 0.012 | 0.020 | 0.061 | 0.056 | 0.022 |  |
| **Time [d]** | **8** | **15** | **21** | **28** | **34** | **42** | **49** | **55** | **62** | **Cell fraction** |
| **Volume Tumor 1** | 0.001 | 0.004 | 0.040 | 0.107 | 0.439 | 0.642 | 0.340 | 1.636 | 1.408 | **CD133^-^** |
| **Volume Tumor 2** | 0.004 | 0.006 | 0.066 | 0.072 | 0.146 | 0.210 | 0.242 | 0.651 | 0.776 |  |
| **Volume Tumor 3** | 0.001 | 0.001 | 0.001 | 0.001 | 0.001 | 0.035 | 0.112 | 0.691 | 0.544 |  |
| **Median volume** | 0.002 | 0.004 | 0.036 | 0.060 | 0.195 | 0.296 | 0.231 | 0.993 | 0.909 |  |
| **sdv** | 0.002 | 0.003 | 0.033 | 0.054 | 0.223 | 0.312 | 0.114 | 0.558 | 0.447 |  |
| **Time [d]** | **8** | **15** | **21** | **28** | **34** | **42** | **49** | **55** | **62** | **Cell fraction** |
| **Volume Tumor 1** | 0.001 | 0.002 | 0.003 | 0.079 | 0.242 | 0.204 | 0.548 | 0.977 | 2.433 | **CD133^+^** |
| **Volume Tumor 2** | 0.001 | 0.001 | 0.001 | 0.006 | 0.006 | 0.006 | 0.366 | 0.773 | 0.855 |  |
| **Volume Tumor 3** | 0.001 | 0.004 | 0.004 | 0.022 | 0.053 | 0.104 | 0.125 | 1.134 | 1.354 |  |
| **Median volume** | 0.001 | 0.002 | 0.003 | 0.036 | 0.100 | 0.105 | 0.346 | 0.961 | 1.547 |  |
| **sdv** | 0.000 | 0.002 | 0.002 | 0.038 | 0.125 | 0.099 | 0.212 | 0.181 | 0.807 |  |
| **p-value*** |  | 0.131 | 0.205 | 0.877 | 0.624 | 0.910 | 0.2701 | 0.0097 | 0.1592 |  |
| **p-value**** | 0.373 | 0.476 | 0.155 | 0.559 | 0.555 | 0.369 | 0.455 | 0.930 | 0.297 |  |
| *unsorted vs. CD133^+^ | | | | | | | | | | |
| **CD133^-^ vs. CD133^+^ | | | | | | | | | | |
